# Small molecule-based detection of non-canonical RNA G-quadruplex structures that modulate protein translation

**DOI:** 10.1093/nar/gkac580

**Published:** 2022-07-08

**Authors:** Yousuke Katsuda, Shin-ichi Sato, Maimi Inoue, Hisashi Tsugawa, Takuto Kamura, Tomoki Kida, Rio Matsumoto, Sefan Asamitsu, Norifumi Shioda, Shuhei Shiroto, Yoshiki Oosawatsu, Kenji Yatsuzuka, Yusuke Kitamura, Masaki Hagihara, Toshihiro Ihara, Motonari Uesugi

**Affiliations:** Division of Materials Science and Chemistry, Faculty of Advanced Science and Technology, Kumamoto University, 2-39-1 Kurokami, Chuo-ku, Kumamoto 860-8555, Japan; Institute for Chemical Research, Kyoto University, Uji, Kyoto 611-0011, Japan; Division of Materials Science and Chemistry, Faculty of Advanced Science and Technology, Kumamoto University, 2-39-1 Kurokami, Chuo-ku, Kumamoto 860-8555, Japan; Graduate School of Science and Technology, Hirosaki University, 3 Bunkyo-cho, Hirosaki, Aomori 036-8561, Japan; Division of Materials Science and Chemistry, Faculty of Advanced Science and Technology, Kumamoto University, 2-39-1 Kurokami, Chuo-ku, Kumamoto 860-8555, Japan; Division of Materials Science and Chemistry, Faculty of Advanced Science and Technology, Kumamoto University, 2-39-1 Kurokami, Chuo-ku, Kumamoto 860-8555, Japan; Division of Materials Science and Chemistry, Faculty of Advanced Science and Technology, Kumamoto University, 2-39-1 Kurokami, Chuo-ku, Kumamoto 860-8555, Japan; Department of Genomic Neurology, Institute of Molecular Embryology and Genetics, Kumamoto University, 2-2-1 Honjo, Chuo-ku, Kumamoto 860-0811, Japan; Department of Genomic Neurology, Institute of Molecular Embryology and Genetics, Kumamoto University, 2-2-1 Honjo, Chuo-ku, Kumamoto 860-0811, Japan; Graduate School of Pharmaceutical Sciences, Kumamoto University, 5-1 Oe, Chuo-ku, Kumamoto 862-0973, Japan; Graduate School of Science and Technology, Hirosaki University, 3 Bunkyo-cho, Hirosaki, Aomori 036-8561, Japan; Division of Materials Science and Chemistry, Faculty of Advanced Science and Technology, Kumamoto University, 2-39-1 Kurokami, Chuo-ku, Kumamoto 860-8555, Japan; Institute for Chemical Research, Kyoto University, Uji, Kyoto 611-0011, Japan; Division of Materials Science and Chemistry, Faculty of Advanced Science and Technology, Kumamoto University, 2-39-1 Kurokami, Chuo-ku, Kumamoto 860-8555, Japan; Graduate School of Science and Technology, Hirosaki University, 3 Bunkyo-cho, Hirosaki, Aomori 036-8561, Japan; Division of Materials Science and Chemistry, Faculty of Advanced Science and Technology, Kumamoto University, 2-39-1 Kurokami, Chuo-ku, Kumamoto 860-8555, Japan; Institute for Chemical Research, Kyoto University, Uji, Kyoto 611-0011, Japan; School of Pharmacy, Fudan University, Shanghai 201203, China

## Abstract

Tandem repeats of guanine-rich sequences in RNA often form thermodynamically stable four-stranded RNA structures. Such RNA G-quadruplexes have long been considered to be linked to essential biological processes, yet their physiological significance in cells remains unclear. Here, we report a approach that permits the detection of RNA G-quadruplex structures that modulate protein translation in mammalian cells. The approach combines antibody arrays and RGB-1, a small molecule that selectively stabilizes RNA G-quadruplex structures. Analysis of the protein and mRNA products of 84 cancer-related human genes identified Nectin*-*4 and CapG as G-quadruplex-controlled genes whose mRNAs harbor non-canonical G-quadruplex structures on their 5′UTR region. Further investigations revealed that the RNA G-quadruplex of CapG exhibits a structural polymorphism, suggesting a possible mechanism that ensures the translation repression in a KCl concentration range of 25–100 mM. The approach described in the present study sets the stage for further discoveries of RNA G-quadruplexes.

## Graphical Abstract

**Graphical Abstract:**
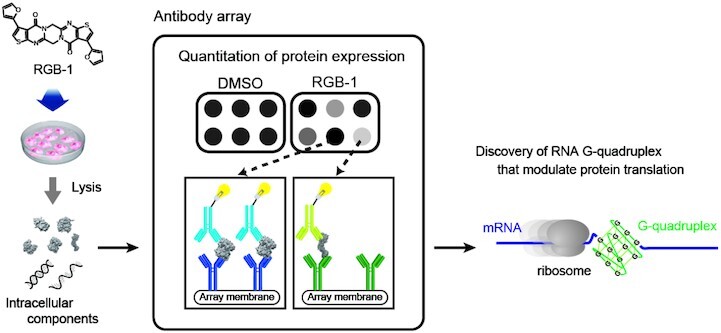
A combined method of antibody arrays and an RNA G-quadruplex stabilizer permits the discovery of uncharacterized, non-canonical RNA G-quadruplex structures that modulate protein translation in a potassium-dependent manner.

## INTRODUCTION

G-quadruplexes are highly stable nucleic-acid structures involved in a myriad of biological processes (1–7). The structures are known to be located at telomeric (1,2,8–10) and promoter regions (3,4,6,7,11) in DNA, modulating genome stability and transcriptional regulation, respectively. A number of small molecules that selectively bind to DNA G-quadruplexes have been developed, providing excellent chemical tools for understanding biological roles and properties of DNA G-quadruplexes (12–17).

RNA G-quadruplexes are thermodynamically more stable than their DNA counterparts (18,19). A number of potential G-quadruplex formation sites on mRNAs have been computationally predicted (20–22). For example, in-silico experiments have predicted 2922 canonical RNA G-quadruplex motifs (G_3_-N_1–7_-G_3_-N_1–7_-G_3_-N_1–7_-G_3_) in the 5′-untranslated regions (5′UTR) in the human genome (23,24). An only fraction of the potential G-quadruplex sites has experimentally been validated (18,19,25–28). RNA G-quadruplex structures have also been found in the internal ribosome entry sites (IRES) (29,30) of mRNAs. However, the physiological significance of these RNA G-quadruplexes remains controversial (31,32). The best-characterized endogenous RNA G-quadruplex is the one located on the 5′UTR of the *NRAS* mRNA (33–36). Deletion of the RNA G-quadruplex-forming sequence from the 5′UTR of *NRAS* mRNA leads to an increase in translational efficiency of the NRAS gene in cells, indicating that the RNA G-quadruplex plays a key role in translational regulation (34,37).

To understand the properties and biological roles of RNA G-quadruplexes in cells, several small-molecule tools selective for RNA G-quadruplexes have been developed (33,38–50). For example, anthrafurandione and anthrathiophenedione molecules bind to RNA G-quadruplexes in the 5′-UTR of *KRAS* oncogene transcript to impair its translation with concomitant anti-cancer properties, exemplifying that RNA G-quadruplexes in oncogene transcripts may serve as drug targets (47). Despite these efforts, specific exogenous control over the cellular roles of RNA G-quadruplexes with a small molecule remain challenging. We previously reported a selective small-molecule RNA G-quadruplex stabilizer, RGB-1, which is capable of repressing the endogenous protein synthesis from RNA G-quadruplex-containing mRNAs in mammalian cells (37). The translational inhibition activity of RGB-1 led us to discover another RNA G-quadruplex latent in the 5′UTR of a proto-oncogene *NRAS* mRNA, raising the possibility that RGB-1 serves as a valuable chemical tool for exploration of functional G-quadruplex-forming sites in a large collection of mRNAs. In the present study, we complement and advance this past work by developing an RGB-1-based approach to search for functional RNA. G-quadruplexes located in the 5′UTR region. The approach described in the present study could pave the way for further discoveries of RNA G-quadruplexes and subsequently a further understanding of the cellular roles of RNA G-quadruplex.

## MATERIALS AND METHODS

### RGB-1

The G-quadruplex stabilizing ligand used in the present study, RGB-1, was originally discovered by chemical library screening in our own laboratory, and its structure was confirmed by chemical synthesis as described in our previous publication (37). The ligand used in the present study was also synthesized in house as described before (37).

### Solubility determination

The solubility of RGB-1 was determined by UV absorption analysis. 1.196, 0.597 and 0.598 mg of RGB-1 were dissolved in 100 μl of DMSO. The UV absorbance of each RGB-1 solution was analyzed by a UV-1650PC (SHIMADZU). *λ*_max_ of RGB-1 in DMSO was estimated to be at 270 nm, and then the molar extinction coefficient (at 270 nm) for RGB-1 was calculated to be 43 100 M^−1^⋅cm^−1^ from the average value. The maximal solubilities of RGB-1 in 1% and 10% DMSO/H_2_O were estimated by measuring the *ϵ_max_* of the solution. The maximal solubilities of RGB-1 in 10% and 1% DMSO/H_2_O were 26 and 11 μM, respectively.

### RNA preparation

RNA samples for UV and CD measurements and footprinting assay were solubilized in 10 mM Tris–HCl (pH 7.5) buffer containing 100 mM KCl or LiCl. Prior to use, the samples were heated to 90°C for 5 min and gradually cooled to room temperature over 60 min in the same buffer.

### Proteome profiler array

The Human XL Oncology Array Kit (R&D Systems) was used for the parallel determination of relative levels of 84 human cancer-related proteins. MCF7 cells were maintained in medium A (Dulbecco's modified Eagle's medium, supplemented with 100 units/ml penicillin, 100 μg/ml streptomycin sulfate, and 10% (v/v) fetal bovine serum) at 37°C in a humidified 5% CO_2_ incubator. On Day 0, MCF7 cells were added to medium A in a 35 mm dish at 4.0 × 10^5^ cells per well. On Day 1, the cells were treated with 1% DMSO (control) or 10 μM RGB-1 in 1% DMSO. After 2 days of culture, the cells were washed three times with cold Phosphate-Buffered Saline (PBS), and lysed with buffer 17 (# 895943, R&D Systems). Blotting assays were performed according to the manufacturer's protocol as follows. Array membranes were treated with 2.0 ml of Array Buffer 6 for 1 h for blocking. After blocking, the membranes were incubated with the cell lysates overnight at 4°C on a rocking platform shaker. Each membrane was washed with 1× Wash Buffer for 10 min for a total of three washes. The membranes were treated with 1.5 ml of Detection Antibody Cocktail and incubated for 1 h. The membranes were treated with 2.0 ml of 1× streptavidin–HRP and Incubated at room temperature for 30 min, and then washed each membrane. After washing, the membranes were treated with 1.0 ml of the Chemi Reagent Mix. and incubated for 1 min. After squeezing out the excess Chemi Reagent Mix, the membranes were placed in an autoradiography film cassette with the identification numbers facing up, and the X-ray film was exposed for 10 min for protein expression analysis.

### Western blot analysis

MCF7 cells were maintained in medium A (Dulbecco's modified Eagle's medium, supplemented with 100 units/ml penicillin, 100 μg/ml streptomycin sulfate, and 10% (v/v) fetal bovine serum) at 37°C in a humidified 5% CO_2_ incubator. On Day 0, MCF-7 cells were added to medium A in a six-well plate at 3 × 10^6^ cells per well. On Day 1, the cells were treated with 1% DMSO (control) or 10 μM RGB-1 in 1% DMSO. After 48 h incubation, the cells were washed three times with cold PBS, and lysed with buffer A [20 mM Tris–HCl (pH 7.5), 150 mM NaCl, 1% (v/v) Nonidet P-40, 0.1% (w/v) sodium deoxycholate, and protease inhibitor cocktail (Nacalai Tesque, Inc.)]. The cell lysates were passed 10 times through a 25G needle and centrifuged at 4°C for 10 min. The supernatants were transferred to new tubes and mixed with 0.20 volume of 6× sodium dodecyl sulfate (SDS) sample buffer (Nacalai Tesque, Inc.). The samples were separated on a 15% sodium dodecyl sulfate poly-acrylamide gel electrophoresis (SDS-PAGE) gel, and blotted using antibodies against CapG (ATFA0420, NKMAX), Nectin-4 (sc-515093, Santa Cruz Biotechnology, Inc.), p53 (sc-126, Santa Cruz Biotechnology, Inc.) or actin (4970, Cell Signaling Technology). The specific bands were visualized using enhanced chemiluminescence (ECL Prime Western Blotting Detection Reagent, GE Healthcare) on an ImageQuant LAS 500 (GE Healthcare).

### Melting temperature (*T*_m_) measurement

The melting temperatures of G-quadruplexes were estimated by monitoring Ultraviolet Visible (UV) absorbance on a UV-1650PC (SHIMADZU) under nitrogen atmosphere, as previously described (51). Temperature scans were performed for G-quadruplexes (5 μM) by scanning continuously from 5 to 95°C at a heating rate of 0.5°C/min at 295 nm in 10 mM Tris–HCl buffer (pH 7.6) containing 2, 4, 6 mM KCl, and data points were collected every 0.5°C. *T*_m_ was set to half the temperature of the maximum signal decrease.

### Circular dichroism (CD) measurement

The CD experiments were performed on a J-725 (JASCO) CD spectrophotometer equipped with a Peltier temperature controller. The spectra were recorded from 220 to 340 nm with a scanning speed of 200 nm/s. This experiment was performed for RNA G-quadruplexes (2.5 μM) in 10 mM Tris–HCl buffer (pH 7.6) containing 100 mM KCl.

### Footprinting assay

Fluorescein (FAM)-labeled RNA oligomers (1.5 μM) were solubilized in 10 mM Tris–HCl (pH 7.5) buffer containing 100 mM KCl or LiCl. The RNA oligomers were ensured to be folded by a heat/cooling process before the subsequent experiments. The RNA samples were treated with 0.05 U/μl RNase T1 (Thermo Fisher Scientific) at room temperature for 3 min, and then a urea-based buffer was added to terminate the reaction. Aliquots of the resulting solution were loaded onto a 15% Tris-borate EDTA (TBE)–urea (7 M) polyacrylamide (acrylamide:bis-acrylamide, 19:1) gel and electrophoresed at 1500 V for 2 h in 1× TBE running buffer. The FAM-labeled RNAs on the gels were visualized using a Typhoon Trio (GE Healthcare).

## RESULTS

### Analysis of 84 cancer-related human genes with RGB-1

**Figure 1.**
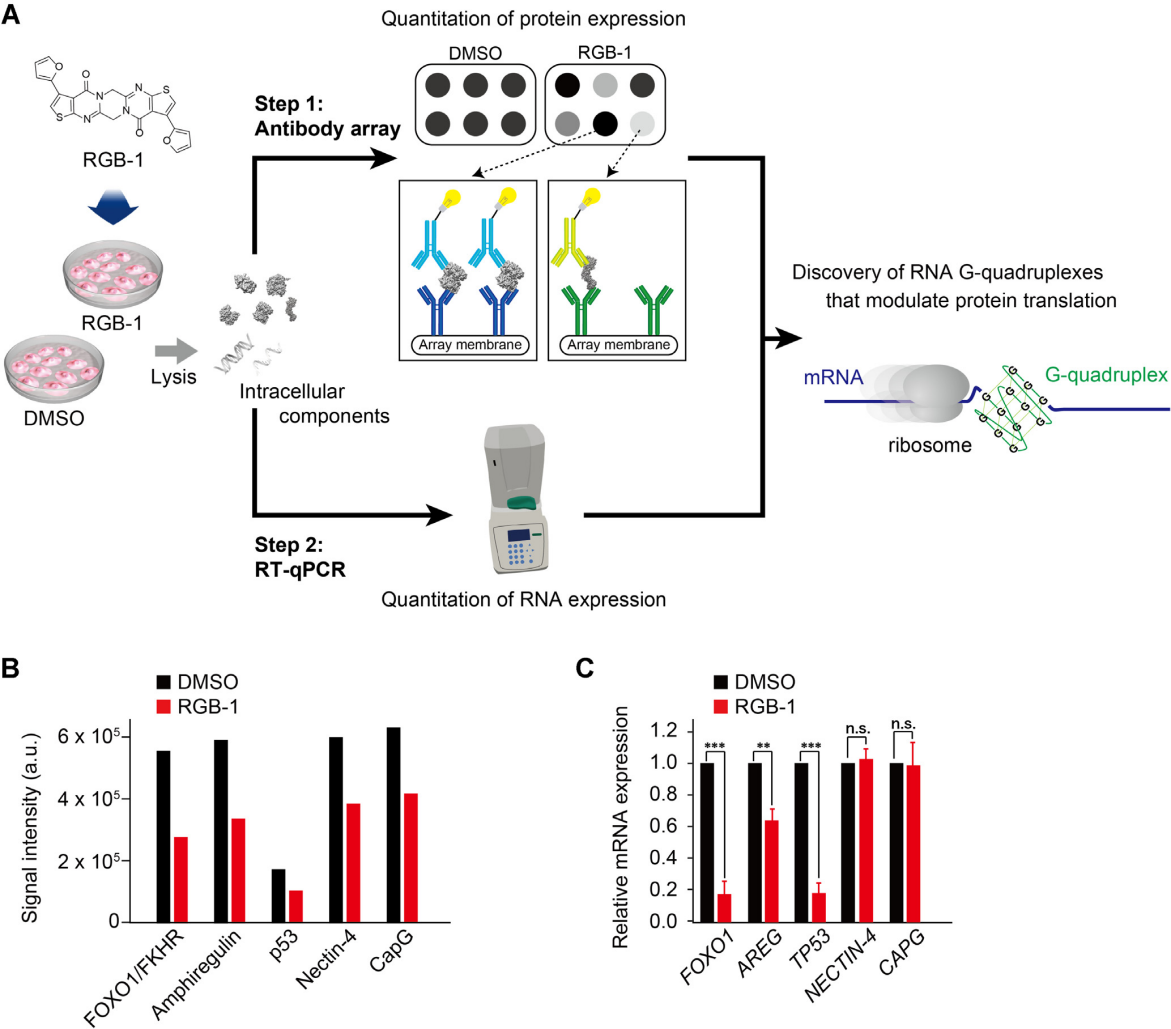
Schematic representation of the strategy to discover intracellular functional RNA G-quadruplexes which affect protein translation reaction. (**A**) Step1: Direct comparison of protein expression levels with an antibody array. The proteins were first extracted from MCF-7 cells which had been treated with RGB-1 or DMSO (the solvent of RGB-1 solution) for 48 h. Each extract was then analyzed by the antibody array. Step2: RT-qPCR analysis of mRNA expression levels of the selected down-regulated gene from the antibody array. The gene candidates selected from Step 1 and Step 2 were further investigated to identify functional RNA G-quadruplexes. (**B**), Comparison of protein expression levels with or without RGB-1 treatment for 5 down-regulated proteins. (**C**) Comparison of RNA expression levels in the presence and absence of RGB-1 for the selected genes. Relative expression level was normalized by GAPDH. Data are shown as mean ± SD. Statistical significance was determined by Student's *t*-test: n.s. = no statistical significance (*P* > 0.05), **P < 0.01 and ***P < 0.001, compared with DMSO alone.

To discover RNA G-quadruplexes in mRNAs, we designed a screening approach in which RGB-1 is employed as an RNA G-quadruplex stabilizer (Figure 1A). The genes encoding the proteins whose expression levels are affected by RGB-1 would theoretically represent or include the genes whose expression is controlled by RNA G-quadruplex formation (Figure 1A). With this hypothesis in mind, human breast cancer MCF-7 cells were treated with DMSO or RGB-1, and protein levels in the cell lysates were analyzed after 48 h. The maximal solubility of RGB-1 in 1%(v/v) DMSO/H_2_O was estimated to be 11 μM. We therefore decided to use 10 μM of RGB-1. To simplify the task of identifying biologically important RNA G-quadruplexes, we focused on cancer-related human genes. Their protein products in the cell lysates were analyzed with an array of antibodies against the protein products of 84 cancer-related human genes. Side-by-side comparison of the blotting data revealed both up-regulated and down-regulated gene products (Figure 1B, Supplementary Figure S1). We focused on the five most down-regulated genes, namely *FOXO1*, *AREG*, *TP53*, *NECTIN-4*, and *CAPG* (Supplementary Tables S1 and S2). The downregulation of the five genes was validated by Western blot analysis (Supplementary Figure S2). We next checked the mRNA expression levels of the five down-regulated genes by quantitative PCR (Figure 1C). The RT-qPCR assays revealed that mRNA expression levels of *FOXO1*, *AREG* and *TP53* were decreased by the RGB-1 treatment in parallel with their protein levels. In contrast, no detectable changes were observed in the mRNA levels of *NECTIN-4* and *CAPG*, although their protein expression levels were significantly reduced. These results suggest that RGB-1 impairs the protein synthesis of Nectin-4 and CapG rather than their mRNA synthesis, prompting us to investigate whether 5′UTRs of Nectin*-*4 and CapG mRNAs harbor any G-quadruplex-forming sequences.

### Identification of RNA G-quadruplex structures

**Figure 2.**
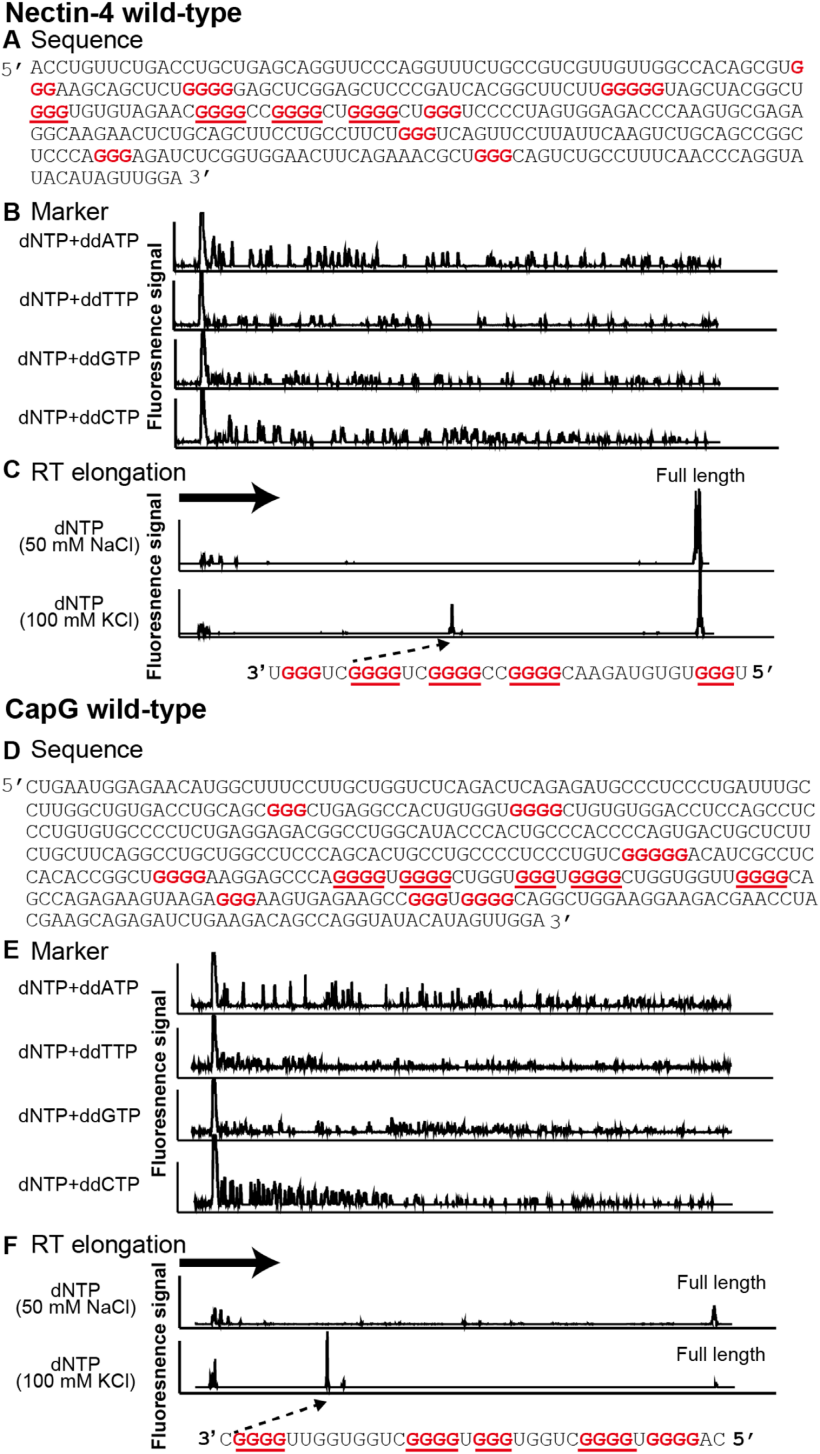
Identification of RNA G-quadruplex formation on Nectin*-*4 wild-type and CapG wild-type mRNAs. (**A, D**) Nucleotide sequences of Nectin*-*4 wild-type and CapG wild-type mRNAs. Guanine repeat sequences (G-tracts) are shown in red. (**B, E**) The fluoresce signals of dNTP + ddATP, dNTP + ddTTP, dNTP + ddGTP and dNTP + ddCTP indicate the A, T, G and C bases on the template DNA strand, respectively. (**C, F**) RTase-mediated cDNA synthesis was interrupted on the Nectin*-*4 wild-type and CapG wild-type mRNAs in the presence of 100 mM KCl, but was not affected in the presence of 50 mM NaCl. G-tracts are shown in red and four G-tract sequences constituting G-quadruplexes are underlined in red. Dashed arrows indicate the arrest sites of reverse transcription. Bold arrows show direction of RTase elongation.

Given the nucleotide sequence of Nectin*-*4 and CapG mRNAs, both of their 5′UTRs are predicted to contain potential G-quadruplex-forming sequences, G_3_-N_1–7_-G_3_-N_1–7_-G_3_-N_1–7_-G_3_. To experimentally locate G-quadruplex sites, we conducted a reverse-transcriptase elongation reaction stop assay (RTase stop assay), in which one can detect the location of RNA G-quadruplex sites as RTase stop signals during the reverse transcription reaction of mRNAs (Supplementary Figure S3) (52,53). In fact, RTase stop signals were detected on the 5′UTRs of both Nectin*-*4 and CapG mRNAs, but not on the 5′UTR of *TP53* mRNA, consistent with the RT-qPCR results. The stop signals were detected only under KCl-containing buffer conditions (Figure 2C, F and Supplementary Figure S4) and further enhanced in the presence of RGB-1 (Supplementary Figure S5). Overall, these results suggested the existence of RNA G-quadruplexes in the 5′UTRs of Nectin*-*4 and CapG mRNA.

The 5′UTR of Nectin*-*4 mRNA contains four guanine tracts (consecutive guanine nucleotides, G-tracts) that are located close to each other. To our surprise, RTase stop assays of its six mutants (mutant1-6) revealed that a G-quadruplex is formed not by the four consecutive G-tracts but by a combination of three of them and a distant G-tract (Figure 2A, C, Supplementary Figure S6 and Supplementary Figure S7). Presumably, the steric configuration of such a separated G-tract combination is more thermodynamically preferable than that of the consecutive G-tracts for building up an RNA G-quadruplex in the Nectin*-*4 mRNA. In fact, replacement of the distant G-tract with an A-tract (mutant7) generated the less preferable G-quadruplex structure of the four consecutive G-tracts (Supplementary Figure S6).

**Figure 3.**
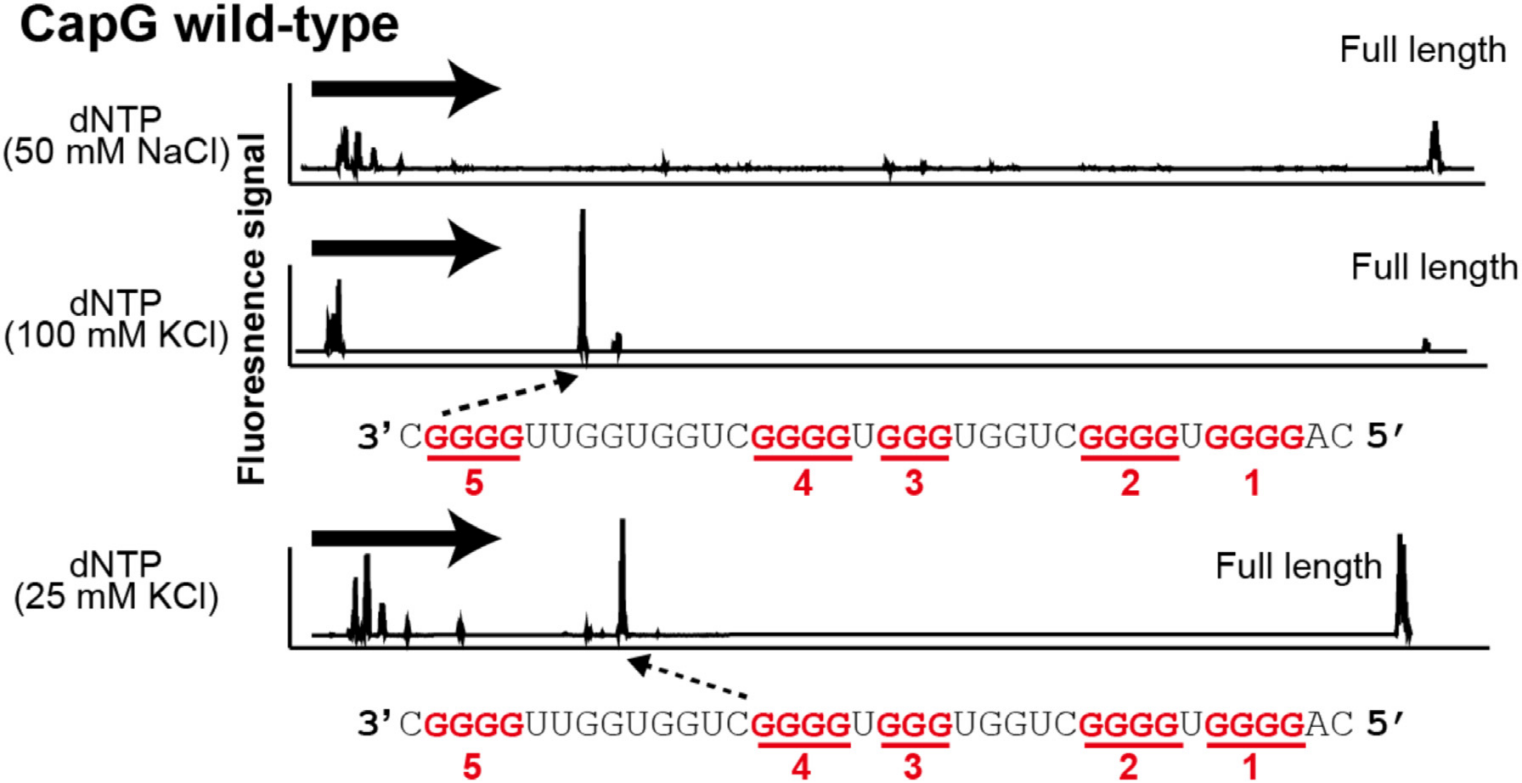
Evaluation of the polymorphic G-quadruplex structure of CapG wild-type. The proportions of each G-quadruplex in the polymorphic structure were evaluated under 50 mM NaCl, 100 mM KCl and 25 mM KCl concentrations by the RTase stop assay. G-tract sequences are shown in red and four G-tract sequences constituting G-quadruplexes are underlined in red. Dashed arrows indicate the arrest sites of reverse transcription. Bold arrows show direction of RTase elongation. The G-tract numbers are shown under the guanine repeat sequences.

On the other hand, the results of the RTase stop assay of the 5′UTR of CapG mRNA, which contains five G-tracts (tracts 1–5), exhibited even more complex patterns (Figure 3). The stop signals under high and low KCl conditions revealed the existence of two RNA G-quadruplex structures, each of which appeared to consist of a distinct set of the four G-tracts: tracts 2–5 and tracts 1–4, respectively. Substitution of tract 1 or 5 with an A-tract (CapG mutant1 or CapG mutant2, respectively) led to the loss of the polymorphic property. These results suggest that the CapG mRNA forms a G-quadruplex of tracts 2–5 at 100 mM KCl and of tracts 1–4 at 25 mM KCl (Supplementary Figure S8).

### Effects of RNA G-quadruplex on protein translation

**Figure 4.**
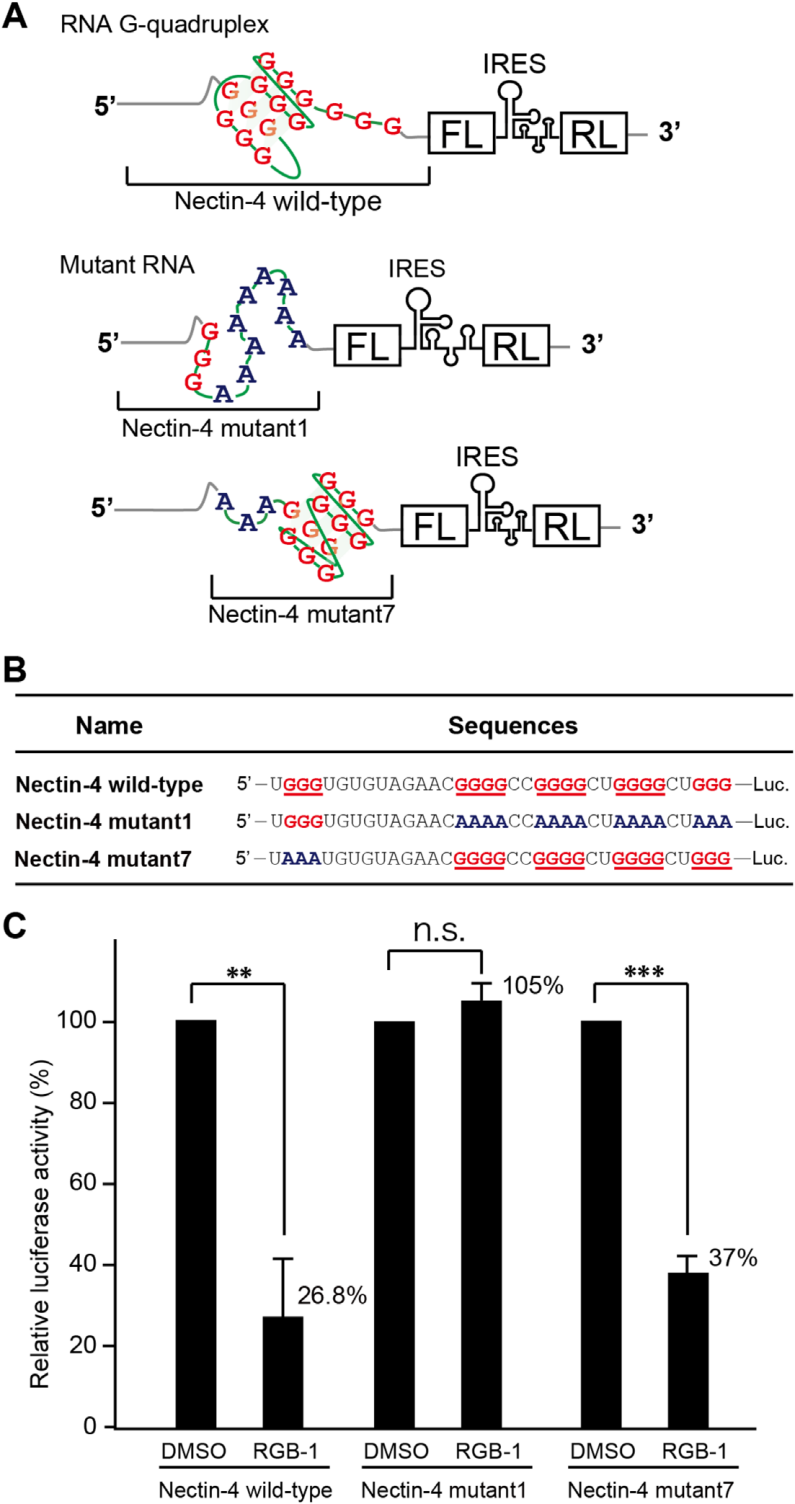
Effect of RGB-1 on translation from the 5′UTR of Nectin*-*4 mRNA. (**A**) The mRNAs encoding firefly luciferase (FL) with a native 5′UTR of Nectin*-*4 and mutated versions of its 5′UTR were designed as models. Renilla luciferase (RL) was placed downstream of an internal ribosomal entry site (IRES) as an internal expression control. (**B**)The nucleotide sequences of the 5′UTRs of the model mRNAs. Luc indicates the firefly luciferase gene. The G-tracts were shown in red. The A-tracts, which were replaced from G-tracts, are shown in blue. (**C**) Effect of RGB-1 on translation of reporter RNA *in vitro*. RGB-1 showed no detectable effect on translation from the non-structured Nectin*-*4 mutant1 RNA template, but, as expected, reduced translation from Nectin*-*4 wild-type and mutant7 RNA templates that contain G-quadruplex structures. Data are shown as mean ± SD. Statistical significance was determined by Student's *t*-test: n.s. = no statistical significance, **P < 0.01 and ***P < 0.001, compared with DMSO alone.

**Figure 5.**
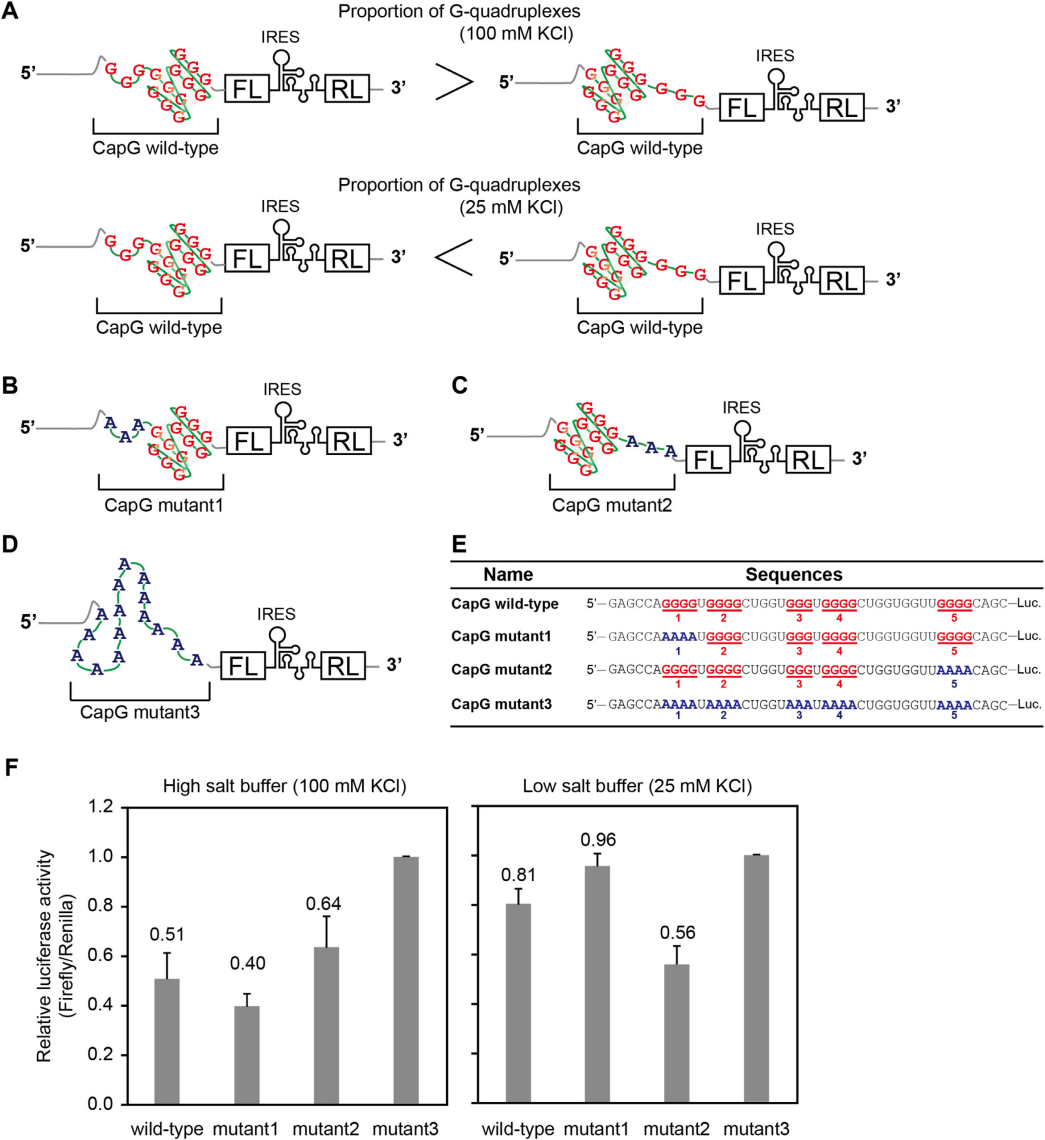
Effects of the polymorphic G-quadruplex structure of CapG 5′UTR on protein synthesis. (**A**) The mRNAs encoding firefly luciferase (FL) with a 5′UTR of CapG wild-type was designed as a model for the evaluation of the effects of the polymorphic G-quadruplex on protein synthesis. Renilla luciferase (RL) was placed downstream of an internal ribosomal entry site (IRES) as an internal expression control. The proportion of the two G-quadruplexes in the polymorphic CapG G-quadruplex depends on KCl concentrations. The tract 2–5 or tract 1–4 are used as the four G-tracts for G-quadruplex formation under 100 mM KCl or 25 mM KCl concentrations, respectively. (**B–D**) The mRNAs encoding FL with 5′UTRs of CapG mutant1, CapG mutant2 and CapG mutant3 were designed as models. RL was placed downstream of an IRES as an internal expression control. (**E**) The nucleotide sequences of the 5′UTRs of the model mRNAs. The G-tracts for G-quadruplex formation are shown in red. The A-tracts, which were replaced from G-tracts, are shown in blue. The five ‘tract’ numbers are shown under the tract sequences. (**F**) Effects of KCl concentration on translation. A high KCl buffer (dilute the translation buffer two thirds times with milli-Q water) and a low KCl buffer (dilute the high KCl buffer 4 times with milli-Q water) were used for in vitro translation. FL activity was normalized to RL activity. Error bars represent standard deviation for at least three independent experiments.

To evaluate the effects of the RNA G-quadruplexes on protein translation, we next used a dual reporter gene in which the translation of Firefly luciferase (FL) is controlled by the 5′UTR of Nectin*-*4 or CapG, while Renilla luciferase (RL) translation is mediated by IRES (Figures 4 and 5) (54). Comparison of the luminescence signals via FL and RL allowed us to quantify the effects of the 5′UTRs. Cell-free translation experiments of the reporter gene revealed that RGB-1 reduced the translational efficiency of the transcripts bearing the wild-type Nectin*-*4 5′UTR, while RGB-1 exhibited no detectable effects on the translation from its mutant that is unable to form a G-quadruplex (mutant1) (Figure 4). On the other hand, mutant7, in which a G-quadruplex is rearranged, maintained its response to RGB-1 (Figure 4). These results are consistent with the notion that the RGB-1-induced translational suppression is mediated by its interaction with the RNA G-quadruplex.

The reporter gene controlled by the CapG 5′UTR displayed an interesting property. RGB-1 similarly reduced the translation of the mRNA bearing wild-type CapG 5′UTR. However, this RGB-1- mediated translation repression was maintained even when either G-tracts 1 or 5 was replaced by an A-tract (CapG mutant1 or 2, respectively). Replacement of both G-tracts 1 and 5 abolished the ability of RGB-1 to repress the FL translation (CapG mutant3) (Supplementary Figure S9). These results suggest that both of the two possible G-quadruplex structures in CapG mRNA contribute to the translational suppression.

The RTase stop assays showed that the proportion of the two CapG G-quadruplexes depends on KCl concentrations (Figure 5A and Supplementary Figure S8). To estimate the impacts of salt concentrations on the translational suppression, we next evaluated the translation efficiency under high (translation buffer: 100 mM KCl) and low (four times diluted translation buffer: 25 mM KCl) salt concentrations using the cell-free translation system. The translation repression activity of CapG mutant1 was as potent as that of CapG wild-type in the high-salt buffer, whereas CapG mutant1 had no significant effects on the translation in the low-salt buffer. In contrast, CapG mutant2 suppressed the translation at the same levels irrespective of the salt concentrations (Figure 5F). Perhaps the concerted pair of the two G-quadruplexes in the CapG 5′UTR ensures the translation repression irrespective of salt concentrations.

### Characterization of G-quadruplex structures

To further characterize the G-quadruplex structures in Nectin*-*4 and CapG, we conducted footprinting. RNase-T1 footprinting experiments depicted clusters of protected guanine residues in the presence of KCl, indicating that these residues are involved in the G-quadruplex formation of Nectin*-*4 and CapG mRNA (Supplementary Figures S10 and S11). The salt-dependent shift of CapG G-quadruplexes was not clearly detected as changes in footprinting signals perhaps due to the conformational equilibrium. Nevertheless, the overall patterns of the protected guanine residues were in good agreement with the results of the RTase stop assays. The G-quadruplex formation was also validated by measuring CD spectra of the RNA oligonucleotides of Nectin*-*4 and CapG. The RNA oligonucleotides encompassing G-quadruplex-forming sites displayed 270 nm positive Cotton effects typical for G-quadruplexes (Supplementary Figure S12). Collectively, these results support our notion that the 5′-UTRs of Nectin*-*4 and CapG form G-quadruplexes.

To corroborate the direct association between RNA G-quadruplexes and RGB-1, we estimated *K*_D_ values of RGB-1 for various G-quadruplex structures using size-exclusion chromatography. The results confirmed that RGB-1 binds selectively to RNA G-quadruplexes, while RGB-1 exhibited no detectable affinity to DNA-DNA or DNA-RNA duplexes (Supplementary Figure S13). The estimated *K*_D_ values for G-quadruplexes for Nectin-4 wild-type, CapG mutant1, and CapG mutant2 were 5.2 ± 1.1, 4.0 ± 1.1 and 3.1 ± 0.6 μM, respectively. These values are comparable with the previously observed affinity of RGB-1 with *TERRA* (*K*_d_ = 5.9 μM) RNA G-quadruplex structure (37).

## DISCUSSION

RNA G-quadruplexes are usually found in the G-rich sequences and have been proposed to play roles in various biological processes encompassing translation (34,37,55,56), splicing (57,58), and transport (59–62). A suite of computational prediction protocols, including G4-RNA Fold (20), cG/cC score (21) and G4Hunter (22), have been reported with the goal of identifying the G-quadruplexes in RNAs, yet most of the predicted G-quadruplexes have not been experimentally confirmed as biologically functional structures. To map RNA G-quadruplexes experimentally, Kwok and Balasubramanian have combined RNA G-quadruplex-mediated reverse transcriptase stalling events with a ligation-amplification strategy and detected RNA G-quadruplex formation in full-length cellular human telomerase RNA (63). The approach described in the present study allowed us to identify two human-cancer-related genes (Nectin*-*4 *and* CapG), whose translation is suppressed by the RNA G-quadruplexes. These two non-canonical yet functional RNA G-quadruplexes have not been precisely detected by the previous computational or experimental studies.

Both Nectin*-*4 and CapG are well-established oncogenes that drive proliferation, metastasis, and angiogenesis in cancerous cells (64,65). Their RNA G-quadruplexes are located in the 5′UTR of the mRNA transcripts, downregulating the translation like those in *NRAS* oncogene (34,37). The 5′UTRs of a number of cancer-linked genes including *MYC*, *MYB*, *NOTCH*, *CDK6* and *BCL2* have been shown to contain 12-nucleotide guanine quartet (CGG)_4_ motif that can form RNA G-quadruplex structures. This motif was identified as a hallmark of RNA transcripts whose translational levels are dependent upon eIF4A RNA helicase, suggesting that RNA G-quadruplexes control eIF4A-dependent oncogene translation (66). The gene repression at the translation stage appears to be a waste of energy for cells. It is not obvious why the 5′UTR of these oncogene transcripts harbor the RNA G-quadruplexes that repress translation.

The secondary or tertiary conformation of RNAs is influenced by salt conditions (25,67,68). Stability of RNA G-quadruplex structures is particularly sensitive to potassium concentrations *in vitro*: higher concentrations usually stabilize RNA G-quadruplex structures (Supplementary Figure S14). Intracellular potassium levels are tightly controlled by potassium channels and vary in cell types: certain efflux potassium channels are overexpressed in a number of cancer cell lines to lower the intracellular potassium levels (69–72). Blockage of the potassium channels in cancer cells results in cell cycle arrest (73,74), suggesting that the high expression of the channels is required for rapid cell proliferation. Sugimoto *et al.* recently proposed that the low intracellular potassium levels in cancer cells decrease the effects of DNA G-quadruplex on gene transcription (75). RNA G-quadruplex may be similarly destabilized by the low potassium levels in cancer cells, such that the translation of G-quadruplex-containing mRNAs is restored to support the growth of cancer cells.

In light of this hypothesis, the CapG G-quadruplex is of particular interests due to its potassium-dependent polymorphic nature. In the case of DNA, such polymorphic G-quadruplexes have been observed to display structural equilibrium in response to salt conditions (25,76–79). For instance, the HIV-1 long terminal repeat (*LTR*) DNA sequence forms a set of two overlapping, interchangeable DNA G-quadruplex structures that regulate gene transcription (80,81). The CapG G-quadruplex represents the first RNA G-quadruplex structure that combines two mutually exclusive G-quadruplex structures exchangeable in response to potassium concentrations. Our results indicate that the structural polymorphism enables the cells to suppress translation of the mRNA both under high and low potassium environments, suggesting its potential role in ensuring translation repression regardless of cellular potassium levels. Further work will be critical for understanding the biological importance of this persistent translational repression of CapG.

In conclusion, our small-molecule-based screening has identified two human cancer-related genes (Nectin*-*4 and CapG), whose translation is suppressed by the RNA G-quadruplexes. These two RNA G-quadruplexes have not been detected by previous computational predictions or experimental screening. Interestingly, the CapG RNA G-quadruplex represents the first RNA G-quadruplex structure that combines two mutually exclusive G-quadruplex structures exchangeable in response to potassium concentrations. Our analysis suggested that this polymorphism plays a role in persistent translation repression. A limitation of our study is that RNA G-quadruplex structures were searched only from the protein products of 84 cancer-related genes. It is unlikely that RNA G-quadruplex structures are unique to oncogenes. A combination of proteome and transcriptome analyses with RGB-1 would permit the quick discovery of uncharacterized, non-canonical RNA G-quadruplexes structures that suppress translation in potassium-dependent and independent manners.

## Supplementary Material

gkac580_Supplemental_File

## Notes

Present address: Kenji Yatsuzuka, Faculty of Pharmaceutical Sciences, Doshisha Women's College of Liberal Arts, Kyotanabe, Kyoto 610-0395, Japan.
