# Supplementary material for: Small molecule-based detection of non-canonical RNA G-quadruplex structures that modulate protein translation
Source: Nucleic Acids Res. 2022 Jul 8;50(14):8143–53. doi: 10.1093/nar/gkac580 (PMC9371906; doi:10.1093/nar/gkac580)
Supplement: gkac580_Supplemental_File [file gkac580_supplemental_file.pdf]

Supporting Information

Small Molecule-Based Detection of Non-Canonical RNA G-Quadruplex Structures That Modulate Protein Translation

Yousuke Katsuda,[†] Shin-ichi Sato,[*§] Maimi Inoue,[†] Hisashi Tsugawa,[∥] Takuto Kamura,[†] Tomoki Kida,[†] Rio Matsumoto,[†] Sefan Asamitsu,[⊥] Norifumi Shioda,[⊥//] Shuhei Shiroto, [∥] Yoshiki Oosawatsu,[†] Kenji Yatsuzuka,[§] Yusuke Kitamura,[†] Masaki Hagihara,[* ∥] Toshihiro Ihara,[*†] Motonari Uesugi[*§‡]

[†]Division of Materials Science and Chemistry, Faculty of Advanced Science and Technology, Kumamoto University, 2-39-1 Kurokami, Chuo-ku, Kumamoto 860-8555, Japan, [⊥]Department of Genomic Neurology, Institute of Molecular Embryology and Genetics, Kumamoto University, [//]Graduate School of Pharmaceutical Sciences, Kumamoto University, Kumamoto, Japan, [§]Institute for Chemical Research, Kyoto University, Uji, Kyoto 611-0011, Japan, [∥]Graduate School of Science and Technology, Hirosaki University, Hirosaki, Aomori 036-8561, Japan, [‡]School of Pharmacy, Fudan University, Shanghai 201203, China

*****E-mail: ssato@scl.kyoto-u.ac.jp (S.S.), hagihara@hirosaki-u.ac.jp (M.H.), toshi@chem.kumamoto-u.ac.jp (T.I.) or uesugi@scl.kyoto-u.ac.jp (M.U.)

Supplementary Contents

Supplementary Methods 3
Supplementary Figures 7
Figure S1. 7
Figure S2. 8
Figure S3. 9
Figure S4. 10
Figure S5. 11
Figure S6. 12
Figure S7. 13
Figure S8. 14
Figure S9. 15
Figure S10. 16
Figure S11. 17
Figure S12 . 18
Figure S13 . 19
Figure S14. 20

Supplementary Tables 21
Table S1. 21
Table S2. 22
Table S3. 23
Table S4. 24

Supplementary Methods

RT-qPCR quantification of five selected gene

MCF7 cells were maintained in medium A at 37 °C in a humidified 5% CO_2 incubator. On Day 0, MCF7 cells were added to medium A in 35 mm dish at 4.0 × 10^5 cells per well. On Day 1, the cells were treated with RGB-1 or DMSO. After 2 day of culture, the cells were harvested by scraping. Total RNA was isolated using ISOGEN (Nippon Gene Co., Ltd.), according to the manufacturer's protocol. First-strand cDNAs were reverse-transcribed from 100 ng samples of the RNA, using an oligo (dT) primer (Eurofins Genomics K.K.) and ReverTra Ace reverse transcriptase (TOYOBO Co.), according to the manufacturer's protocol. The cDNAs were subjected to qPCR, using a pair of *GAPDH* primers to quantify *GAPDH* mRNA [5'-GCA CCG TCA AGG CTG AGA AC-3' and 5'-TGG TGA AGA CGC CAG TGG A-3'], a pair of primers to quantify *FOXO1* mRNA [5'-GCA ACG CGT GGG GCA ACC TGT-3' and 5'-GGG CAC GCT CTT CAC CAT CCA CTC-3'], a pair of primers to quantify *AREG* mRNA [5'-GGC CAT TAT GCT GCT GGA T-3' and 5'-TGT GGT CCC CAG AAA ATG GT-3'], a pair of primers to quantify *TP53* mRNA [5'-ACT CTG TCT CCT TCC TCT TCC TAC AG-3' and 5'-GTG TGG AAT CAA CCC ACA GCT-3'], a pair of primers to quantify *NECTIN-4* mRNA [5'-GCC TTG AAG ACC AAA AGC TG-3' and 5'-CAA CCG TGT CCA GTT GTA CG-3'], and a pair of primers to quantify *CAPG* mRNA [5'-ACA CAG CCA TTC CCC AGA GT-3' and 5'- AGT CCC CCG AGA AGA AGA CG-3']. The qPCR analysis was performed on a MiniOpticon™ Real-Time PCR System (Bio-Rad Laboratories, Inc.) with SYBR® Green (TAKARA BIO INC.).

Preparation of the Nectin-4, CapG, *TP53* 5'UTR and reverse transcriptase (RTase) stop assay

The human Nectin-4 5'UTR fragment wild-type was amplified by PCR from genomic DNA (Novagen) with a specific primer set [5'- TAA TAC GAC TCA CTA TAG ACC TGT TCT GAC CTG CTG AGC AGG TTC CCA GGT TTC-3'] and [5'- TCC AAC TAT GTA TAC CTG GGT TGA AAG GCA GAC TGC CCA GCG TTT CTG AAG TTC-3'] and cloned into pMD19 vector (pMD19-Nectin-4 wild-type-5'UTR construct). The human CapG 5'UTR fragment wild-type was amplified by PCR from genomic DNA (Novagen) with a specific primer set [5'- TAA TAC GAC TCA CTA TAG CTG AAT GGA GAA CAT GGC TTT CCT TGC TGG TCT CA-3'] and [5'- TCC AAC TAT GTA TAC CTG GCT GTC TTC AGA TCT CTG CTT CGT AGG TTC GTC TTC CTT CCA GCC TGC -3'] and cloned into pMD19 vector (pMD19-CapG wild-type-5'UTR construct). Mutants of CapG 5'UTR (mutant1) were prepared by overlap extension PCR. Two DNA fragments were amplified by PCR, using the pMD19-CapG wild-type-5'UTR constructs, with a forward primer [5'-CGG TAC CCG GGG ATC TAA TAC GAC TCA CTA TAG CTG -3'] and a reverse primer [5'-CCA GCC CCA TTT TTG GGC TCC-3'], or a forward primer [5'-CCC AAA AAT GGG GCT GGT GGG TGG GGC TGG TGG-3'] and a reverse primer [5'- CGA CTC TAG AGG ATC TCC AAC TAT GTA TAC CTG GCT GTC TTC AGA TCT CTG C -3']. These PCR products were connected by overlap extension PCR, using a forward primer [5'- CGG TAC CCG GGG ATC TAA TAC GAC TCA CTA TAG CTG -3'] and a reverse primer [5'- CGA CTC TAG AGG ATC TCC AAC TAT GTA TAC CTG GCT GTC TTC AGA TCT CTG C -3']. The PCR products were subcloned into the *Bam*H I site in pUC19 vector by using an In-Fusion HD cloning kit (TAKARA BIO INC.) (pUC19-CapG mutant1-5'UTR construct). Mutants of CapG 5'UTR (mutant2) were prepared by overlap extension PCR. Three DNA fragments (Section1, Section2, and Section3) were amplified by PCR. Section1 was

amplified by PCR, using the pMD19-CapG wild-type-5'UTR constructs, with a forward primer [5'-CGG TAC CCG GGG ATC TAA TAC GAC TCA CTA TAG CTG -3'] and a reverse primer [5'-CCG GTG TGG AGG CGA TGT CCC CCG ACA GGG-3']. Section2 was amplified by PCR, using a synthetic oligo-nucleotide-template [5'- CTC CAC ACC GGC TGG GGA AGG AGC CCA GGG GTG GGG CTG GTG GGT GGG GCT GGT GGT TAA AAC AGC CAG AGA AGT AAG A-3'], forward primer [5'-CCC TGT CGG GGG ACA TCG CCT CCA CAC CGG C-3'] and reverse primer [5'-CTC ACT TCC CTC TTA CTT CTC TGG CTG-3']. Section3 was amplified by PCR, using the pMD19-CapG wild-type-5'UTR constructs, a forward primer [5'-CAG CCA GAG AAG TAA GAG GGA AGT GAG-3'] and a reverse primer [5'- CGA CTC TAG AGG ATC TCC AAC TAT GTA TAC CTG GCT GTC TTC AGA TCT CTG C -3']. Each set of PCR products Section1 and Section2, or Section2 and Section3 was connected by overlap extension PCR, using a forward primer [5'-CGG TAC CCG GGG ATC TAA TAC GAC TCA CTA TAG CTG -3'] and a reverse primer [5'-CTC ACT TCC CTC TTA CTT CTC TGG CTG-3'], or a forward primer [5'-CCC TGT CGG GGG ACA TCG CCT CCA CAC CGG C-3'] and a reverse primer [5'- CGA CTC TAG AGG ATC TCC AAC TAT GTA TAC CTG GCT GTC TTC AGA TCT CTG C -3'], respectively. These PCR products were connected by overlap extension PCR, using a forward primer [5'- CGG TAC CCG GGG ATC TAA TAC GAC TCA CTA TAG CTG -3'] and a reverse primer [5'- CGA CTC TAG AGG ATC TCC AAC TAT GTA TAC CTG GCT GTC TTC AGA TCT CTG C -3']. The PCR products were subcloned into the *Bam*H I site in pUC19 vector by using the In-Fusion HD cloning kit (pUC19-CapG mutant2-5'UTR construct). Mutants of CapG 5'UTR (mutant3) were prepared by overlap extension PCR. Three DNA fragments (Section1, Section2, and Section3) were amplified by PCR. Section1 was amplified by PCR, using the pMD19-CapG wild-type-5'UTR constructs, with a forward primer [5'-CTA GCC TCG AGA ATT CTG AAT GGA GAA CAT GGC TTT CC-3'] and a reverse primer [5'-CCG GTG TGG AGG CGA TGT CCC CCG ACA GGG-3']. Section2 was amplified by PCR, using the synthetic oligo-nucleotide-template [5'- CTC CAC ACC GGC TGG GGA AGG AGC CCA AAA ATA AAA CTG GTA AAT AAA ACT GGT GGT TAA AAC AGC CAG AGA AGT AAG A-3'], a forward primer [5'-CCC TGT CGG GGG ACA TCG CCT CCA CAC CGG C-3'] and a reverse primer [5'-CTC ACT TCC CTC TTA CTT CTC TGG CTG-3']. Section3 was amplified by PCR, using the pMD19-CapG wild-type-5'UTR constructs, a forward primer [5'-CAG CCA GAG AAG TAA GAG GGA AGT GAG-3'] and a reverse primer [5'-CCA TGG TGG CGA ATT GCT GTC TTC AGA TCT CTG C-3']. Each set of PCR products Section1 and Section2, or Section2 and Section3 was connected by overlap extension PCR, using a forward primer [5'- CTA GCC TCG AGA ATT CTG AAT GGA GAA CAT GGC TTT CC-3'] and a reverse primer [5'-GAC GGC CAG TGA ATT CTG AAT GGA GAA-3'], or a forward primer [5'-CCC TGT CGG GGG ACA TCG CCT CCA CAC CGG C-3'] and a reverse primer [5'- CCA TGG TGG CGA ATT GCT GTC TTC AGA TCT CTG C-3'], respectively. These PCR products were connected by overlap extension PCR, using a forward primer [5'- CTA GCC TCG AGA ATT CTG AAT GGA GAA CAT GGC TTT CC-3'] and a reverse primer [5'- CCA TGG TGG CGA ATT GCT GTC TTC AGA TCT CTG C-3']. The PCR products were subcloned into the *Bam*H I site in pUC19 vector by using the In-Fusion HD cloning kit (pUC19-CapG mutant3-5'UTR construct). The human *TP53* 5'UTR fragment wild-type was amplified by PCR from genomic DNA (Novagen) with a specific primer set [5'- TAA TAC GAC TCA CTA TAG ACT TGT CAT GGC GAC TGT CCA GCG TGT CAC CGT CGT GGA AAG C -3'] and cloned into pMD19 vector (pMD19 *TP53* wild-type 5'UTR construct). Each dsDNA for RNA transcription were prepared from each constructs by PCR-amplification with a T7 promoter primer [5'-TAA TAC GAC TCA CTA TAG CTG AAT GGA GAA CAT GGC TTT CCT TGC TGG TCT CA-3'] and a 3'-DNA primer [5'-TCC AAC TAT GTA TAC CTG GCT GTC

TTC AGA TCT CTG CTT CGT AGG TTC GTC TTC CTT CCA GCC TGC C-3']. The dsDNAs were transcribed to ssRNA using a T7 RNA Polymerase (New England Biolabs). The RNAs were purified with After Tri Reagent RNA Clean Up Kit (Chiyoda Science Co., ltd.). A reaction mixture of template RNA (0.3 μM), a primer [5'-FAM- TCC AAC TAT GTA TAC CTG -3'] (0.1 μM), and KCl or NaCl buffer (Tris-HCl pH 8.0, DTT, and KCl or NaCl) were heated to 80 °C for 3 min and cooled to ambient temperature. ReverTra Ace reverse transcriptase (TOYOBO), $MgCl_2$, and dNTPs were then added to the reaction mixture, and the samples were heated to 42 °C for 90 min and 99 °C for 5 min. The reaction products were purified and analyzed on an ABI3500 capillary DNA Sequencer (Life Technologies Japan Ltd.).

Preparation of the Nectin-4 wild-type and Nectin-4 mutant and *in vitro* translation assay.

The dsDNA for Renilla luciferase (RL) was amplified by PCR using psiCHECK-2 vector (Promega) as a template, with a forward primer [5'- TCG ACC CGG GCG GCC ATG GCT TCC AAG GTG TAC G-3'] and a reverse primer [5'- TAA AGG GAA GCG GCC TTA CTG CTC GTT CTT CAG C-3']. The PCR products were subcloned into the *Not* I site in pIRES vector (Clontech Laboratories, Inc.) by using the In-Fusion HD cloning kit (pIRES-RL construct). The dsDNA for firefly luciferase (FL) was amplified by PCR using psiCHECK-2 vector as a template, with a forward primer [5'-CTA GCC TCG AGA ATT CGC CAC CAT GGC CGA TGC TAA GAA C-3'] and a reverse primer [5'-CTC GAC GCG TGA ATT TTA CAC GGC GAT CTT GCC-3']. The PCR products were subcloned into the *Eco*R I site in pIRES-RL constructs by using the In-Fusion HD cloning kit (pIRES-FL-RL construct). The dsDNA for Nectin-4 wild-type was amplified by PCR using pMD19-Nectin-4 wild-type construct as a template, with a forward primer [5'- CTA GCC TCG AGA ATT ACC TGT TCT GAC CTG CTG -3'] and a reverse primer [5'- CCA TGG TGG CGA ATT TCC AAC TAT GTA TAC CTG GG -3']. The PCR products were subcloned into the *Eco*R I site in pIRES-FL-RL constructs by using the In-Fusion HD cloning kit. Nectin-4 mutants were prepared by overlap extension PCR. Three DNA fragments (Section1, Section2, and Section3) were amplified by PCR. Section1 was amplified by PCR, using pMD19-Nectin-4 wild-type construct as a template, with a forward primer [5'- CTA GCC TCG AGA ATT ACC TGT TCT GAC CTG CTG -3'] and a reverse primer [5'- CTA CAC ACC CAG CCG TAG CTA CC -3']. Section2 was amplified by PCR, using synthetic oligo-nucleotide-template [5'- GTG TAG AAC AAA ACC AAA ACT AAA ACT AAA TCC CCT AGT G-3'], a forward primer [5'- GGT AGC TAC GGC TGG GTG TGT AGA AC -3'] and a reverse primer [5'- CGC ACT TGG GTC TCC ACT AGG GG -3']. Section3 was amplified by PCR, using pMD19-Nectin-4 wild-type construct as a template, with a forward primer [5'- CCC CTA GTG GAG ACC CAA GTG C-3'] and a reverse primer [5'- CCA TGG TGG CGA ATT TCC AAC TAT GTA TAC CTG GG-3']. These PCR products were connected by overlap extension PCR, using a forward primer [5'- CTA GCC TCG AGA ATT ACC TGT TCT GAC CTG CTG -3'] and a reverse primer [5'- CCA TGG TGG CGA ATT TCC AAC TAT GTA TAC CTG GG -3']. The PCR products were subcloned into the *Eco*R I site in pIRES-FL-RL construct by using the In-Fusion HD cloning kit. The linear dsDNA templates for RNA transcription were amplified by PCR using the pIRES constructs as templates, with a forward primer [5'- TAA TAC GAC TCA CTA TAG GGC TAG CCT CGA G -3'] and a reverse primer [5'- CTC GAC GCG TGA ATT TTA CAC GGC GAT CTT GCC -3']. The PCR products were separated and purified by 1% agarose electrophoresis, followed by gel extraction. RNAs were transcribed from the dsDNA templates using T7 RNA polymerase, and purified using a NAP-5 column (GE Healthcare Life Sciences) to remove unincorporated NTPs. The resulting Nectin-4 wild and mutant type, were used as mRNA templates (1 μg) in 20 μL of cell-free protein expression mixture (RTS 100 Wheat Germ CECF Kit, 5 Prime, Inc.), with or without 10 μM of RGB-1, for 1 h at 24 °C. Luciferase activity was

evaluated using the Luciferase Assay kit (Promega) and a POWERSCAN H1 microplate reader (BioTek).

Preparation of the CapG wild-type, CapG mutant1, CapG mutant2 and CapG mutant3 and *in vitro* translation assay.

The dsDNA for CapG wild-type, CapG mutant1, and CapG mutant2 were amplified by PCR using pMD19-CapG wild-type-5'UTR, pUC19-CapG mutant1-5'UTR and pUC19-CapG mutant2-5'UTR constructs as templates, with a forward primer [5'-CTA GCC TCG AGA ATT CTG AAT GGA GAA CAT GGC TTT CC-3'] and a reverse primer [5'- CCA TGG TGG CGA ATT GCT GTC TTC AGA TCT CTG C -3']. The PCR products were subcloned into the *Eco*R I site in pIRES-FL-RL constructs by using the In-Fusion HD cloning kit. The CapG mutant3 was prepared by overlap extension PCR. Three DNA fragments (Section1, Section2, and Section3) of mutant3 were amplified by PCR. Section1 was amplified by PCR, using the pMD19-CapG wild-type-5'UTR construct as a template, with a forward primer [5'- CTA GCC TCG AGA ATT CTG AAT GGA GAA CAT GGC TTT CC -3'] and a reverse primer [5'-CCG GTG TGG AGG CGA TGT CCC CCG ACA GGG-3']. Section2 was amplified by PCR, using the synthetic oligo-nucleotide-template [5'- CTC CAC ACC GGC TGG GGA AGG AGC CCA AAA ATA AAA CTG GTA AAT AAA ACT GGT GGT TAA AAC AGC CAG AGA AGT AAG A -3'], forward primer [5'-CCC TGT CGG GGG ACA TCG CCT CCA CAC CGG C-3'] and reverse primer [5'-CTC ACT TCC CTC TTA CTT CTC TGG CTG-3']. Section3 was amplified by PCR, using the pMD19-CapG wild-type-5'UTR construct as a template, with a forward primer [5'-CAG CCA GAG AAG TAA GAG GGA AGT GAG-3'] and a reverse primer [5'-CCA TGG TGG CGA ATT GCT GTC TTC AGA TCT CTG C-3']. Each sets of PCR products of Section1 and Section2, or Section2 and Section3 were connected by overlap extension PCR, using a forward primer [5'-CTA GCC TCG AGA ATT CTG AAT GGA GAA CAT GGC TTT CC-3'] and a reverse primer [5'-CTC ACT TCC CTC TTA CTT CTC TGG CTG-3'], or a forward primer [5'-CCC TGT CGG GGG ACA TCG CCT CCA CAC CGG C-3'] and a reverse primer [5'- CCA TGG TGG CGA ATT GCT GTC TTC AGA TCT CTG C -3'], respectively. These PCR products were connected by overlap extension PCR, using a forward primer [5'- CTA GCC TCG AGA ATT CTG AAT GGA GAA CAT GGC TTT CC-3'] and a reverse primer [5'- CCA TGG TGG CGA ATT GCT GTC TTC AGA TCT CTG C -3']. The PCR products were subcloned into the *Eco*R I site in pIRES-FL-RL constructs by using the In-Fusion HD cloning kit. The linear dsDNA templates for RNA transcription were amplified by PCR using the pIRES constructs as templates, with a forward primer [5'- TAA TAC GAC TCA CTA TAG GGC TAG CCT CGA G-3'] and a reverse primer [5'- TAA AGG GAA GCG GCC TTA CTG CTC GTT CTT CAG C-3']. The PCR products were separated and purified by 1% agarose electrophoresis, followed by gel extraction. RNAs were transcribed from the dsDNA templates using T7 RNA polymerase, and purified using the NAP-5 column to remove unincorporated NTPs. The resulting CapG wild-type, mutnant1, mutnant2, and mutnant3, were used as mRNA templates (1 μg) in 20 μL of cell-free protein expression mixture, with or without 10 μM of RGB-1, for 1 h at 24 °C. Luciferase activity was evaluated using a Luciferase Assay kit (Promega) and a POWERSCAN H1 microplate reader (BioTek).

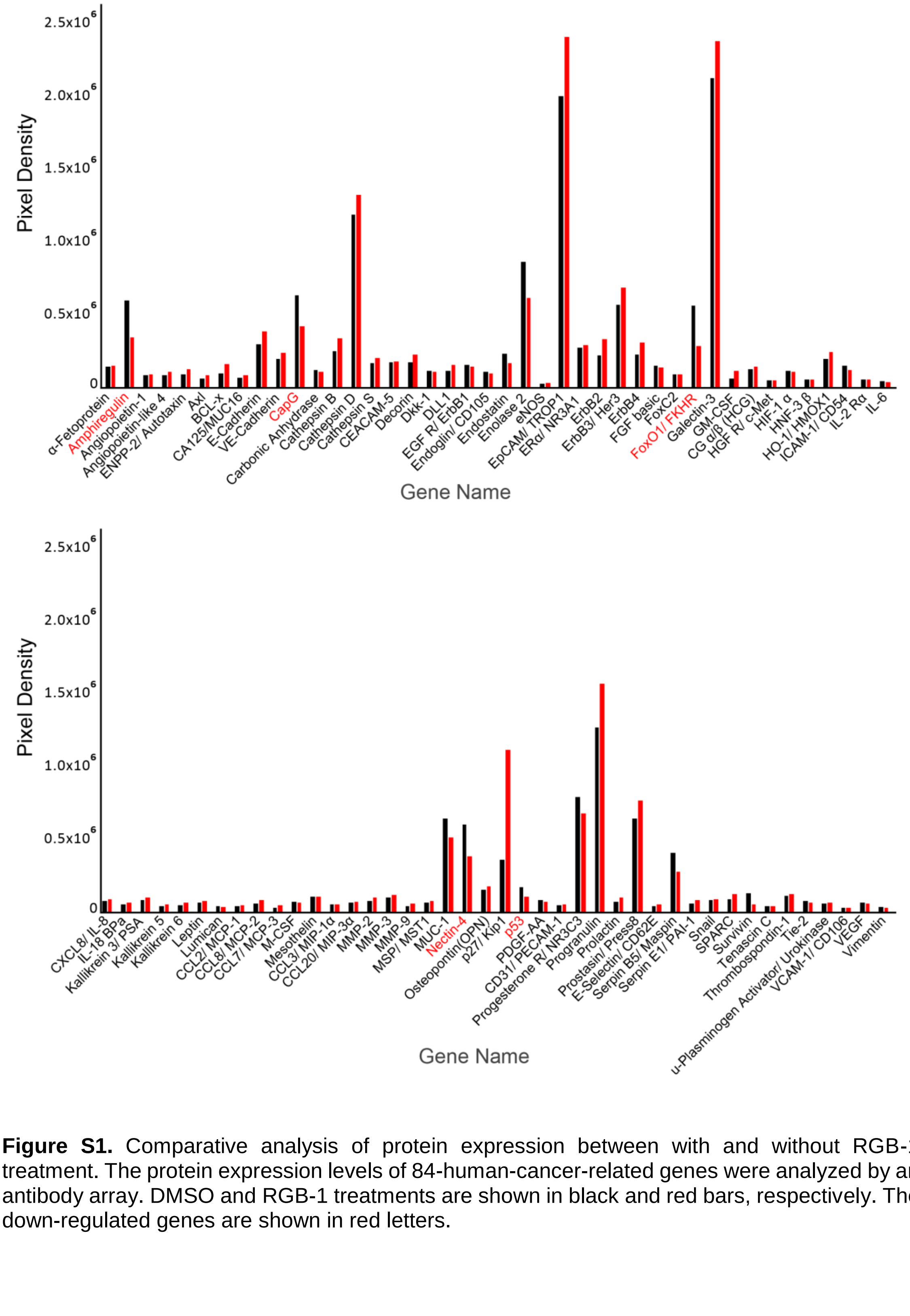

Figure S1. Comparative analysis of protein expression between with and without RGB-1 treatment. The protein expression levels of 84-human-cancer-related genes were analyzed by an antibody array. DMSO and RGB-1 treatments are shown in black and red bars, respectively. The down-regulated genes are shown in red letters.

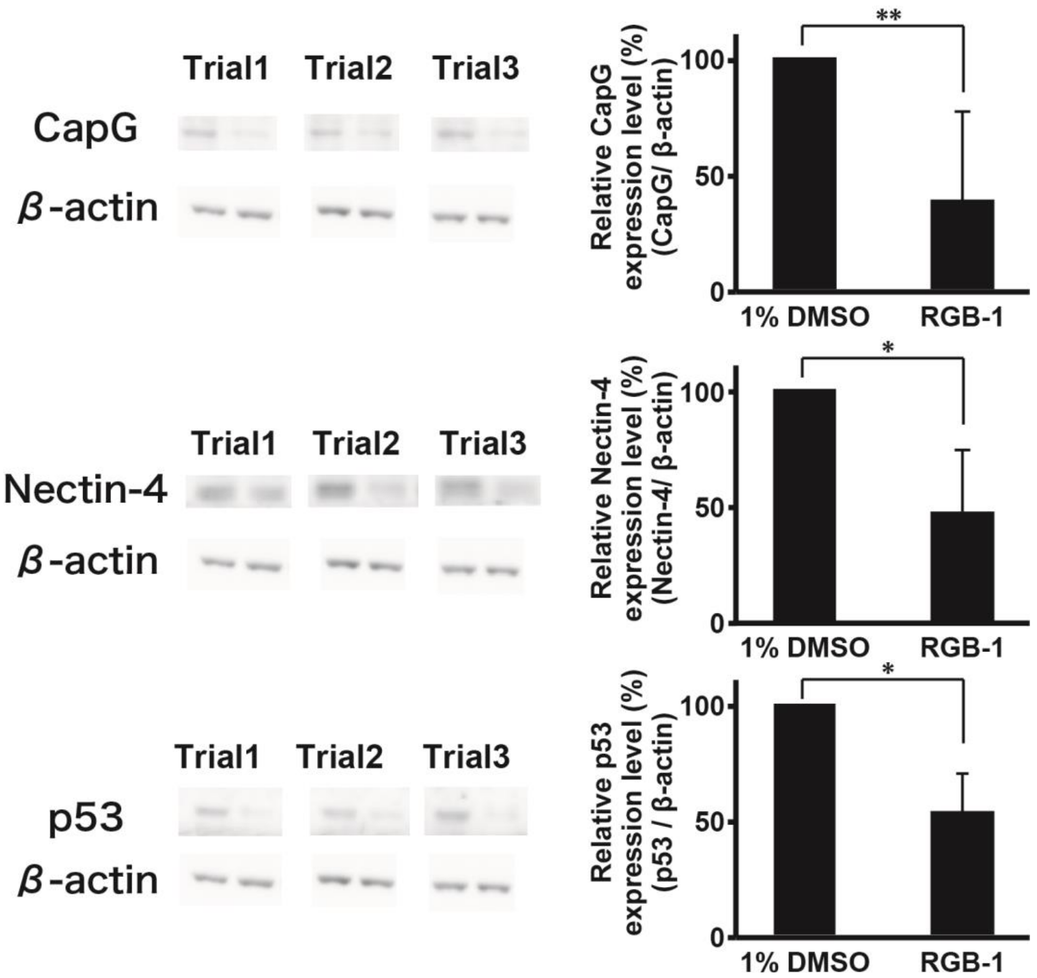

Figure S2. Effects of RGB-1 on the protein levels of CapG, Nectin-4, and p53. MCF7 cells were treated with 10 μM RGB-1 for 48 h. (*Left panel*) The cell lysates were immunoblotted with the indicated antibodies. β-actin was used as a loading control. w/o indicates DMSO control. The results of three independent experiments for each protein are shown (1-5). (*Right panel*) Quantification of the western blot results. Data are shown as mean ±SD. Statistical significance was determined by Student’s t-test: *P<0.05, and **P<0.01, compared with DMSO alone.

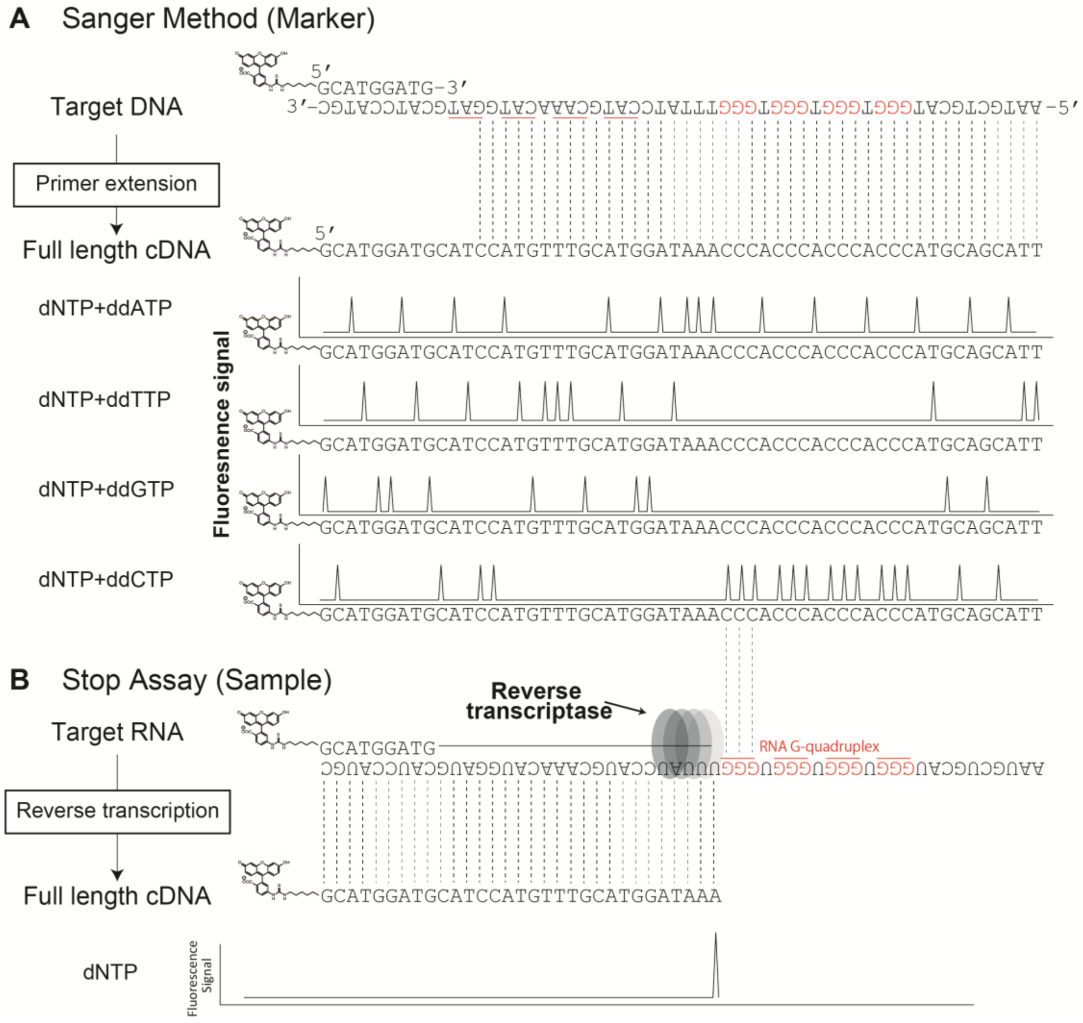

Figure S3. Graphical presentation of RTase stop assays for identification of RNA G-quadruplex. **A,** The Sanger method was used to prepare sequencing markers. **B,** In RTase stop assays, a fluorescently labeled oligonucleotide primer and a reverse transcriptase with a mixture of deoxynucleotide triphosphates (normal dNTPs) were used for extension of complementary DNA strands. The presence of an RNA G-quadruplex arrests the elongation of complementary DNA strand, resulting in a positive detection signal.

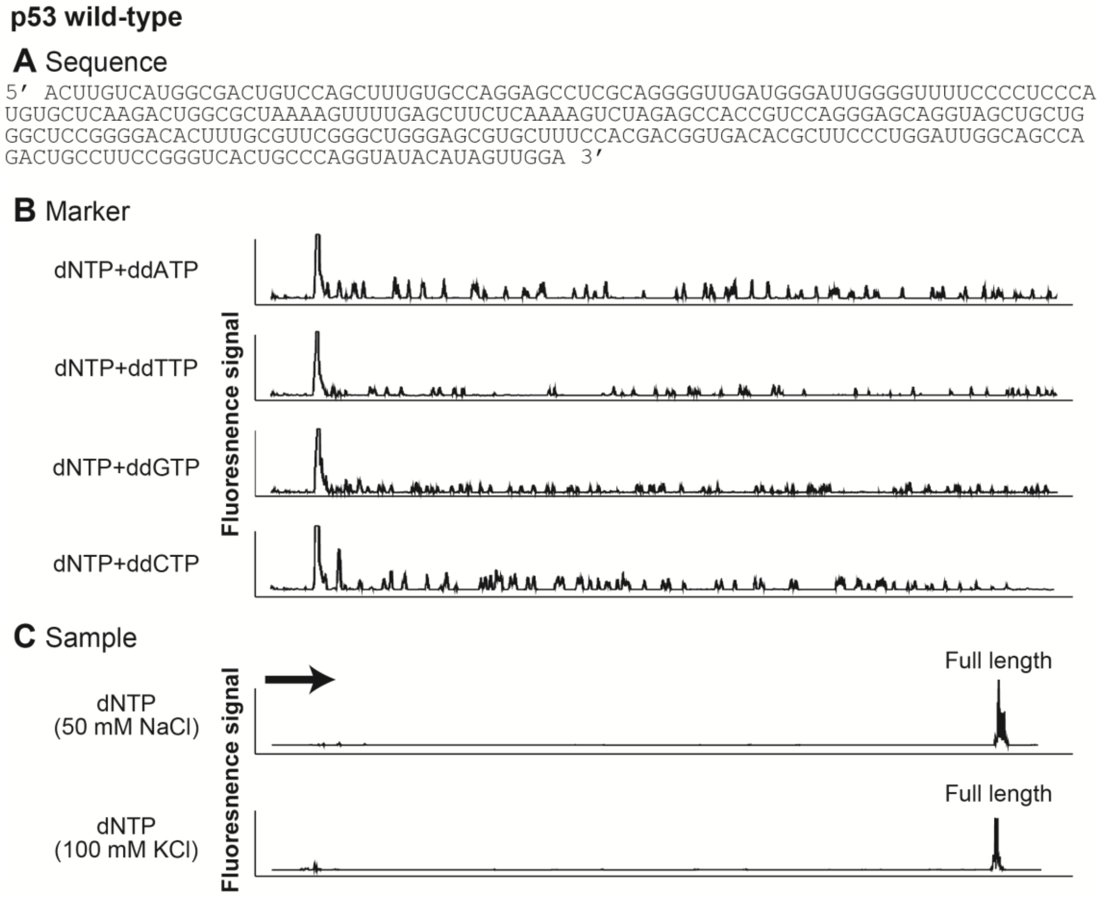

Figure S4. RTase stop assays of the 5’-UTR of *TP53* mRNA. **A,** Nucleotide sequence of the *TP53* 5’-UTR. **B,** The fluorescent signals of dNTP+ddATP, dNTP+ddTTP, dNTP+ddGTP and dNTP+ddCTP indicate the A, T, G and C bases on the template DNA strand, respectively. **C,** No detectable RTase stop signals were observed in the presence of 100 mM KCl and 50 mM NaCl. Bold arrows show the direction of RTase elongation.

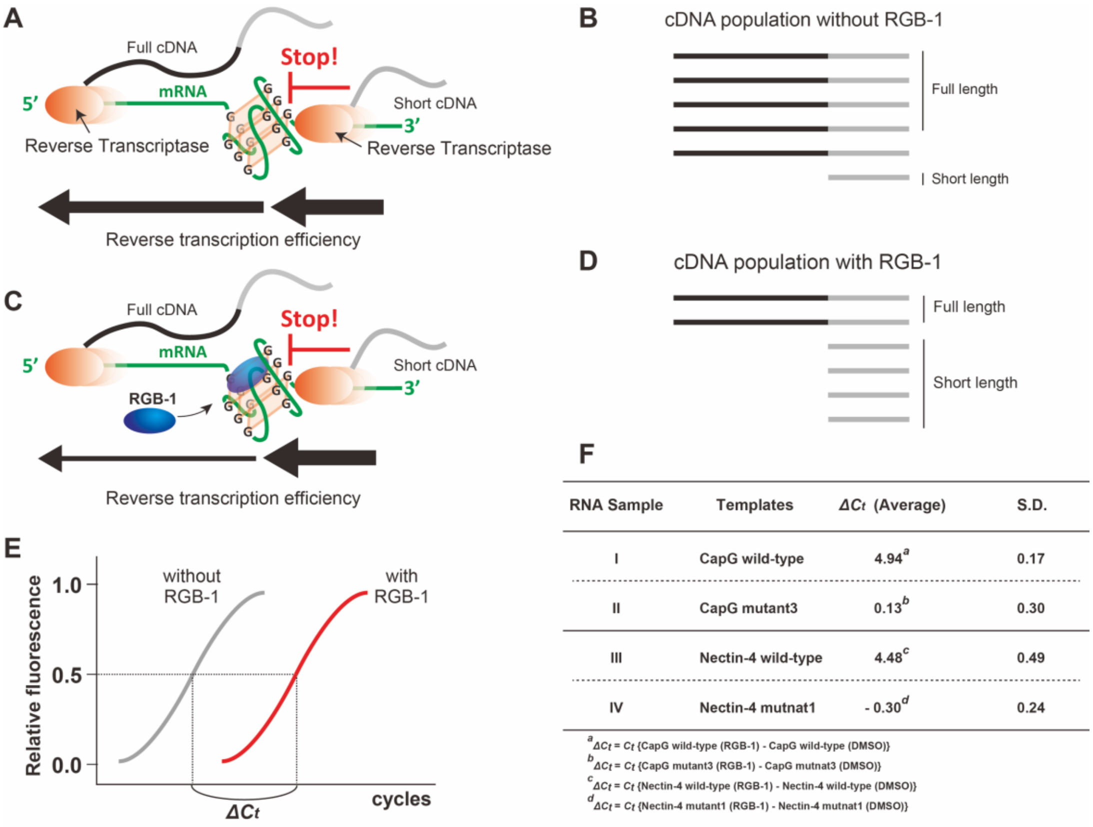

RNA Sample	Templates	ΔC_t (Average)	S.D.
I	CapG wild-type	4.94[a]	0.17
II	CapG mutant3	0.13[b]	0.30
III	Nectin-4 wild-type	4.48[c]	0.49
IV	Nectin-4 mutnat1	- 0.30[d]	0.24

[a] $\Delta C_t = C_t$ {CapG wild-type (RGB-1) - CapG wild-type (DMSO)}
[b] $\Delta C_t = C_t$ {CapG mutant3 (RGB-1) - CapG mutnat3 (DMSO)}
[c] $\Delta C_t = C_t$ {Nectin-4 wild-type (RGB-1) - Nectin-4 wild-type (DMSO)}
[d] $\Delta C_t = C_t$ {Nectin-4 mutant1 (RGB-1) - Nectin-4 mutnat1 (DMSO)}

Figure S5. RT-PCR-based RNA G-quadruplex detection using RGB-1. **A-E,** Graphical presentation of the detection method. **A, B,** The presence of an RNA G-quadruplex partially blocks the elongation reaction of reverse transcriptase, producing both full- and short-length cDNA populations. **C, D,** RGB-1 arrests reverse transcription at the site of G-quadruplex, resulting in reduced production of full-length cDNA. The amounts of the full-length cDNA products are quantified by quantitative PCR (qPCR). **E,** ΔC_t values are determined by subtracting the C_t value of qPCR with RGB-1 from that without RGB-1. The higher ΔC_t value indicates the more potent inhibition of reverse transcription. **F,** The ΔC_t values in qPCR for 5'UTR of Nectin-4 and CapG mRNA. RGB-1 reduced the production of full-length cDNA from RNA templates containing Nectin-4 and CapG 5'UTR (CapG wild-type and Nectin-4 wild-type), but not from the RNA templates in which G-tracts were mutated (CapG mutant3 and Nectin-4 mutant1).

A Sequence

5′ ACCUGUUCUGACCUGCUGAGCAGGUUCCCAGGUUUCUGCCGUCGUUGUUGGCCACAGCGU**GGG**A
AGCAGCUCU**GGGG**GAGCUCGGAGCUCCCGAUCACGGCUUCUU**GGGGG**UAGCUACGGCU**GGG**UGU
GUAGAAC**GGGG**CC**GGGG**CU**GGGG**CU**GGG**UCCCCUAGUGGAGACCCAAGUGCGAGAGGCAAGAAC
UCUGCAGCUUCCUGCCUUCU**GGG**UCAGUUCCUUAUUCAAGUCUGCAGCCGGCUCCCA**GGG**AGAU
CUCGGUGGAACUUCAGAAACGCU**GGG**CAGUCUGCCUUUCAACCCAGGUAUACAUAGUUGGA 3′

B

Name	Sequences
**Nectin-4 mutant2**	5’– GU**GGG**AAGCAGCUCU**GGGG**GAGCUCGGAGCUCCCGAUCACG<br>GCUUCUU**GGGGG**UAGCUACGGCU**GGG**UGUGUAGAAC**GGGG**C<br>C**GGGG**CU**GGGG**CU**AAA**UC–3’
**Nectin-4 mutant3**	5’– GU**GGG**AAGCAGCUCU**GGGG**GAGCUCGGAGCUCCCGAUCACG<br>GCUUCUU**GGGGG**UAGCUACGGCU**AAA**UGUGUAGAAC**GGGG**C<br>C**GGGG**CU**GGGG**CU**AAA**UC–3’
**Nectin-4 mutant4**	5’– GU**GGG**AAGCAGCUCU**GGGG**GAGCUCGGAGCUCCCGAUCACG<br>GCUUCUU**AAAAA**UAGCUACGGCU**GGG**UGUGUAGAAC**GGGG**C<br>C**GGGG**CU**GGGG**CU**AAA**UC–3’
**Nectin-4 mutant5**	5’– GU**GGG**AAGCAGCUCU**AAAA**GAGCUCGGAGCUCCCGAUCACG<br>GCUUCUU**GGGGG**UAGCUACGGCU**GGG**UGUGUAGAAC**GGGG**C<br>C**GGGG**CU**GGGG**CU**AAA**UC–3’
**Nectin-4 mutant6**	5’– GU**AAA**AAGCAGCUCU**GGGG**GAGCUCGGAGCUCCCGAUCACG<br>GCUUCUU**GGGGG**UAGCUACGGCU**GGG**UGUGUAGAAC**GGGG**C<br>C**GGGG**CU**GGGG**CU**GGG**UC–3’
**Nectin-4 mutant7**	5’– GU**GGG**AAGCAGCUCU**GGGG**GAGCUCGGAGCUCCCGAUCACG<br>GCUUCUU**GGGGG**UAGCUACGGCU**AAA**UGUGUAGAAC**GGGG**C<br>C**GGGG**CU**GGGG**CU**GGG**UC–3’

C

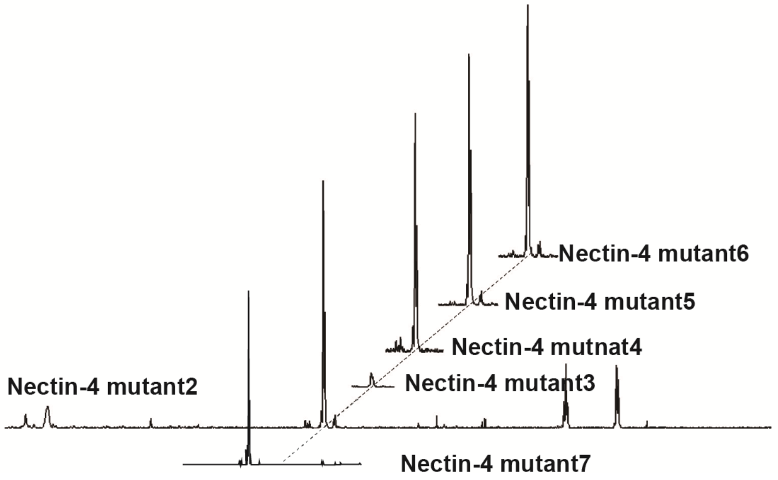

Figure S6. RTase stop assays for five Nectin-4 mutants. **A,** Nucleotide sequence of the Nectin-4 5’UTR. Guanine repeat sequences (G-tracts) are shown in red. The four G-tracts that are involved in the G-quadruplex formation of Nectin-4 are underlined. **B,** The nucleotide sequences of the Nectin-4 mutants. The A-tracts, which replaced the G-tracts, are shown in blue. **C,** The Nectin-4 mutant3 displayed no detectable stop signals, whereas the other mutants exhibited inhibition of RTase-mediated cDNA synthesis at their G-quadruplex-forming sites. These results indicate that the four G-tracts underlined in **A,** are main components of the Nectin-4 RNA G-quadruplex.

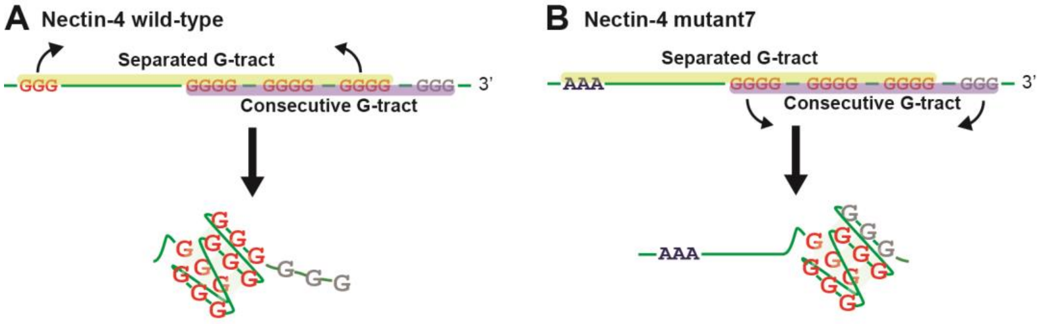

Figure S7. The schematic diagrams of the possible G-quadruplex structures in both wild and mutant7 types of Nectin-4 RNA G-quadruplex. **A,** The G-quadruplex is formed not by the four consecutive G-tracts (purple) but by a combination of three of them and a distant G-tract (yellow) in Nectin-4 wild-type. **B,** Nectin-4 mutant7 forms an RNA G-quadruplex with the consecutive G-tract because it cannot select the distant G-tract for RNA G-quadruplex formation.

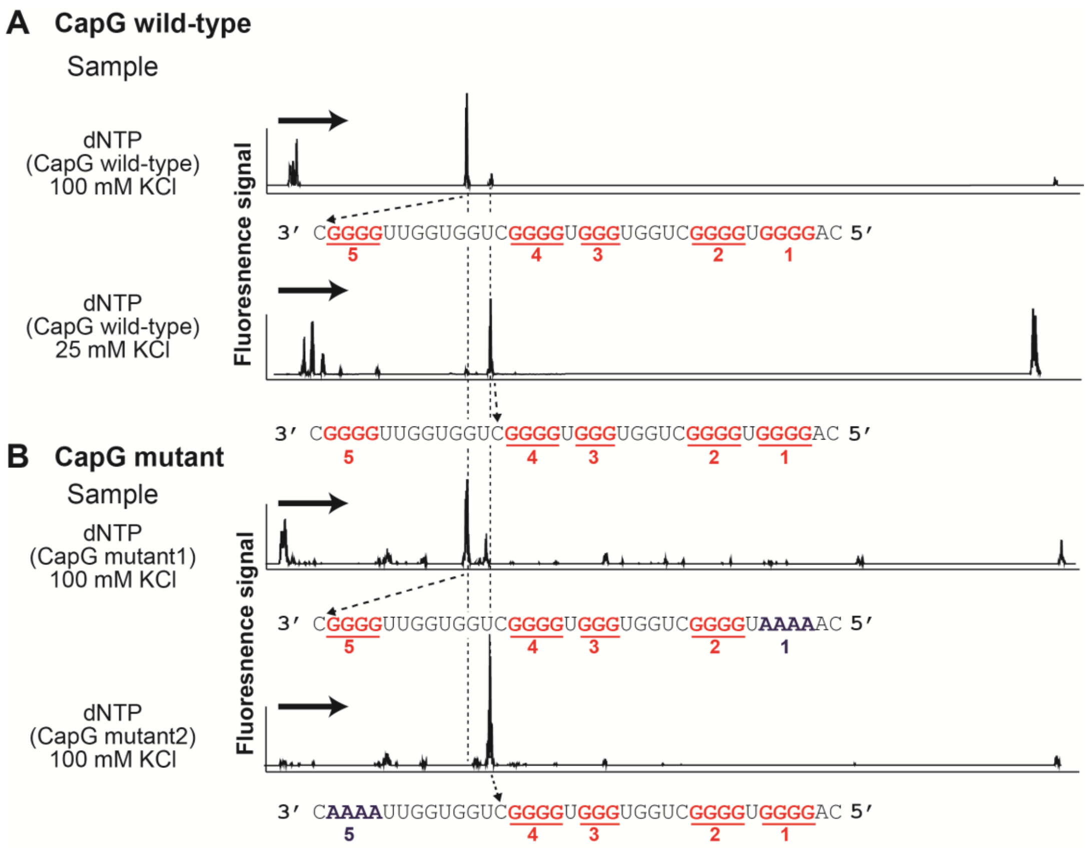

Figure S8. RTase stop assay for CapG wild-type, CapG mutant1, and CapG mutant2. **A,** Comparison of the RTase stop signals in the 5'UTR of CapG wild-type in the presence of 100 mM or 25 mM KCl. **B,** The RTase stop signals in the 5'UTR of CapG mutant 1 or mutant2 in the presence of 100 mM KCl. The G-tract sequences are shown in red. The four G-tracts involved in G-quadruplex formation are underlined. The A-tracts, which replaced G-tracts, are shown in blue. Dashed arrows indicate the arrest sites of reverse transcription. Bold arrows show direction of the elongation reaction of reverse transcriptase. The G-tract numbers are shown under the guanine repeat sequences.

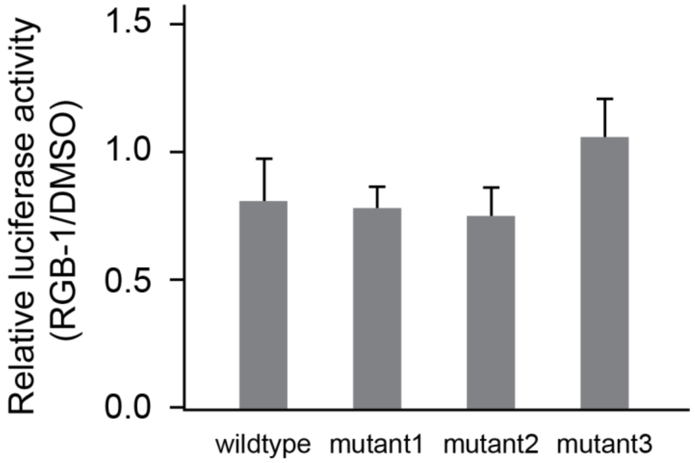

Figure S9. Effects of RGB-1 on translation of luciferase reporter mRNAs controlled by the 5’UTR of CapG. The mRNAs encoding firefly luciferase (FL) with a native 5’UTR of CapG and two mutated version of its 5’UTR were designed as models. Renilla luciferase (RL) was placed downstream of an internal ribosomal entry site (IRES) as an internal expression control. RGB-1 (10 µM) showed no detectable effects on protein translation from the reporter mRNA with CapG mutant3, while RGB-1 suppressed translation from the reporter mRNAs with CapG wild-type, CapG mutant1, or CapG mutant2.

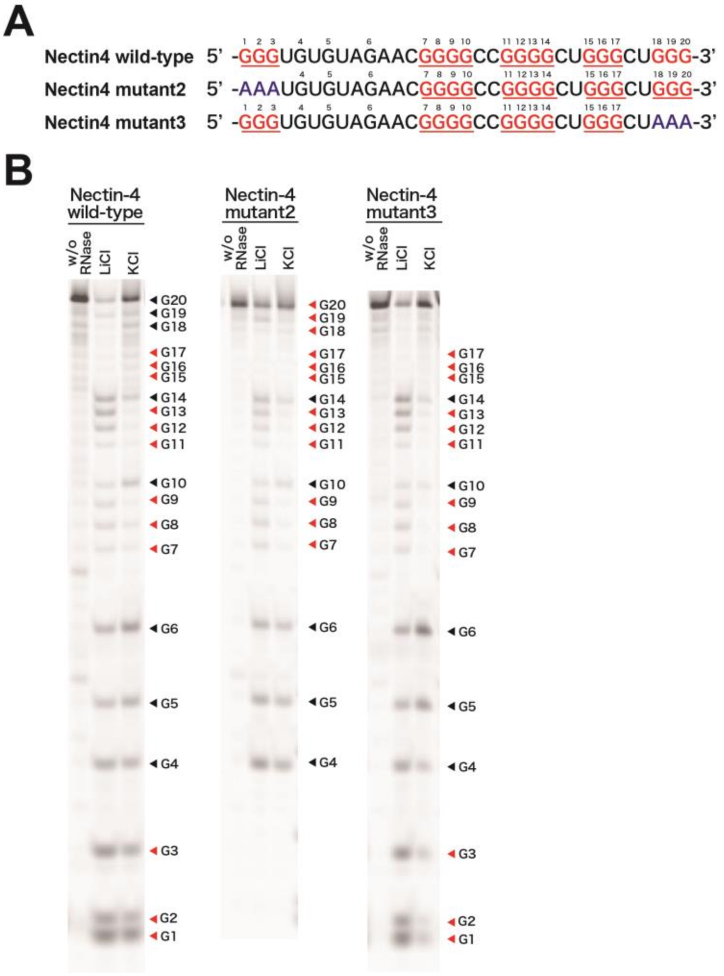

Figure S10. RNase T1 footprinting experiments showing protection of Nectin-4 G-quartet-forming guanines from RNase T1 cleavage. **A,** Nucleotide sequences of the 5'FAM labeled oligonucleotides (Nectin-4 wild type, mutant2, and mutant3). Identities of the guanine residues are indicated by numbering. **B,** Each of the FAM-labeled RNA oligonucleotides was treated with RNase T1 in the presence of a LiCl or KCl-containing buffer. The RNA samples were loaded and run on a polyacrylamide gel. The positions of the guanine residues are indicated on the right side. The guanines protected by KCl from RNase T1-mediated cleavage are indicated with red arrow heads.

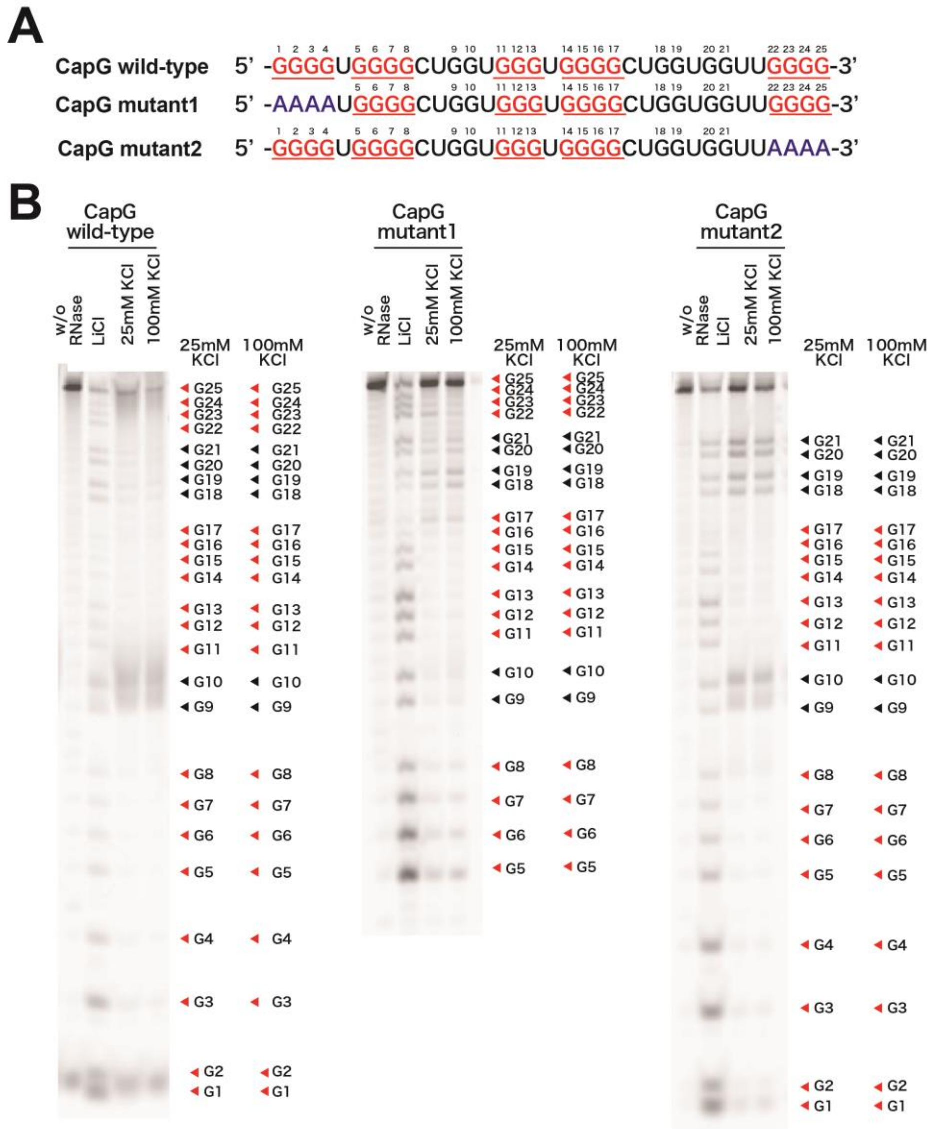

Figure S11. RNase T1 footprinting experiments showing protection of CapG G-quartet-forming guanines from RNase T1 cleavage. **A,** Nucleotide sequences of the 5'FAM labeled oligonucleotides (CapG wild type, mutant1, and mutant2). Identities of the guanine residues are indicated by numbering. **B,** Each of the FAM-labeled RNA oligonucleotides was treated with RNase T1 in the presence of a LiCl or KCl (25 or 100 mM)-containing buffer. The RNA samples were loaded and run on a polyacrylamide gel. The positions of the guanine residues are indicated on the right side. The guanines protected by KCl from RNase T1-mediated cleavage are indicated by red arrow heads.

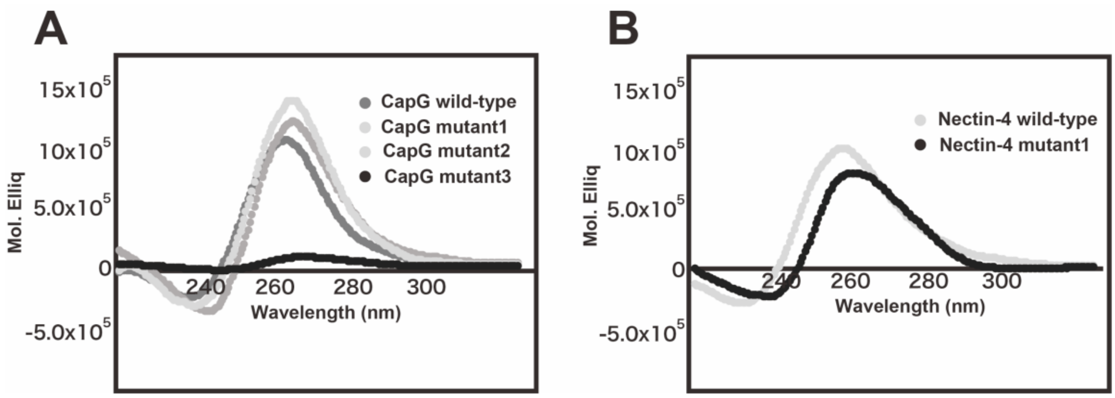

Figure S12. Circular dichroism (CD) spectra of **A,** CapG and **B,** Nectin-4 oligonucleotides. The sequences of the wild-type and mutant RNA oligonucleotides are shown in Table S4. The spectra were recorded from 220 to 340 nm with a scanning speed of 200 nm/s. The experiment was performed for each RNA oligonucleotide (2.5 µM) in 10 mM Tris-HCl buffer (pH 7.6) containing 100 mM KCl.

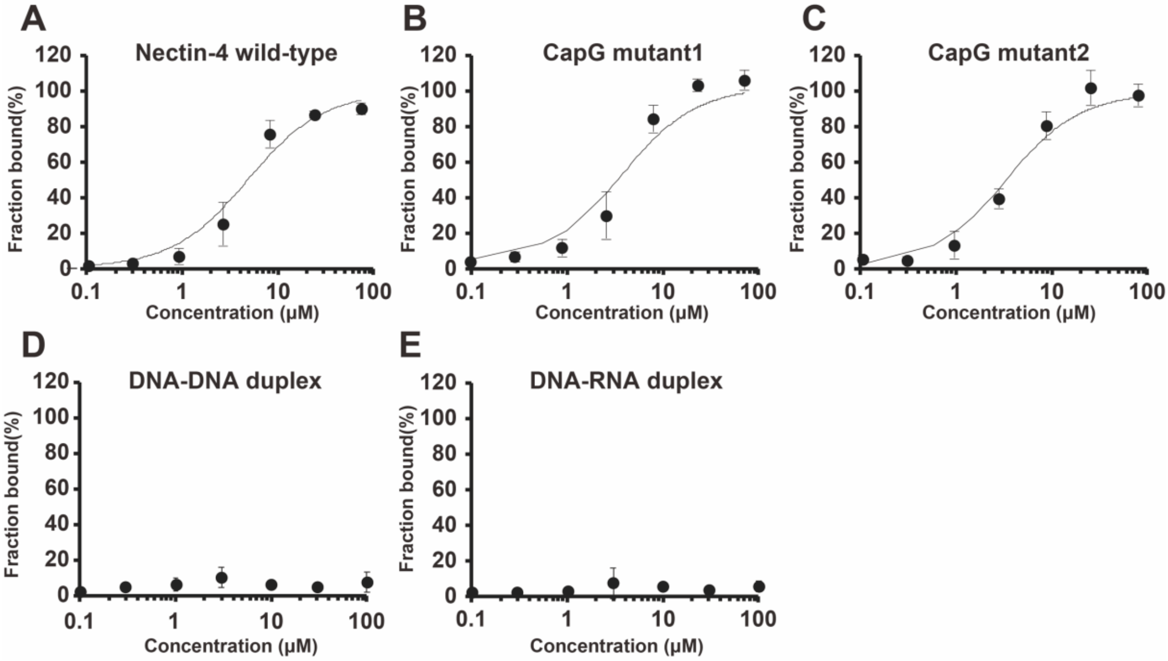

Figure S13. Semilogarithmic plots showing the fraction of RGB-1 bound to G-quadruplex-forming oligonucleotides (**A,** Nectin-4 wild-type; **B,** CapG mutant1; **C,** CapG mutant2; **D,** DNA-DNA duplex; and **E,** DNA-RNA duplex). RGB-1 was incubated in binding buffer (10 mM Tris-HCl (pH 7.6) containing 100 mM KCl) at room temperature in the presence of oligonucleotides (0, 0.1, 0.3, 1, 3, 10, 30, or 100 μM). The oligonucleotide sequences used in the study are shown in **Table S4.** After a 30-minute incubation, the reaction mixture was separated by gel filtration using illustra™ MicroSpin™ G-25 columns. The flow-through fraction was collected and treated with RNase A or DNase to degrade oligonucleotides. The fraction was then subjected to HPLC for quantification of RGB-1. HPLC analyses were normalized to the peak area of theophylline, co-injected as an internal standard. Error bars represent standard deviation for at least three independent experiments.

A

Name	Sequences
shCapG mutant1	5'–AAAAUGGGGCUGGUGGGUGGGGCUGGUGGUUGGGG–3'<br>1 2 3 4 5
shCapG mutant2	5'–GGGGUGGGGCUGGUGGGUGGGGCUGGUGGUUAAAA–3'<br>1 2 3 4 5

B

KCl concentration	2 mM	4 mM	6 mM
shCapG mutant1	55.0 °C	61.1 °C	64.6 °C
shCapG mutant2	66.1 °C	70.6 °C	73.1 °C

C

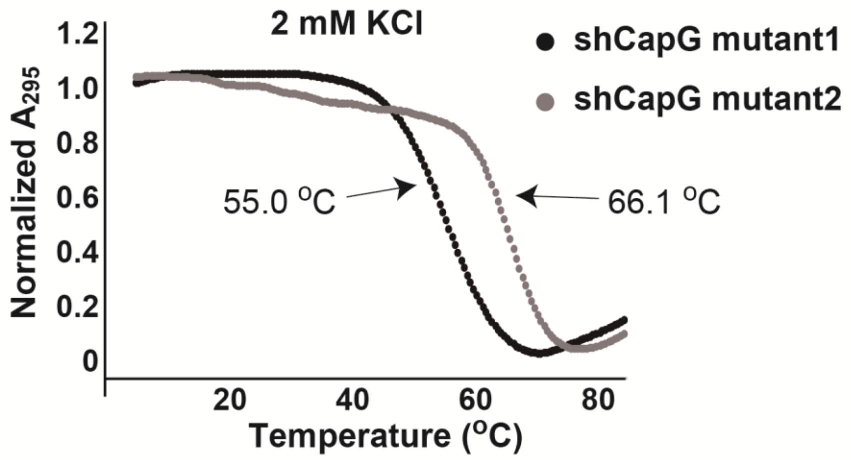

Figure S14. T_m measurement for short version of CapG mutant1 and mutant2. **A,** Nucleotide sequences of shCapG mutant1 and shCapG mutant2. The four G-tracts for G-quadruplex formation of mutants are shown in red. The A-tracts, which replaced G-tracts, are shown in blue. The five "tract" numbers are shown under the repeat sequences. **B,** Melting temperatures of shCapG mutant1 and mutant2 under various KCl concentrations. **C,** Comparison of melting curves of shCapG mutant1 (black circles) and shCapGmutant2 (gray circles) under 2 mM KCl concentrations.

Table S1. Protein expression levels of 84 cancer-related human genes. Blotting raw data are shown.

Analyte	DMSO(Control)	RGB-1
α-Fetoprotein	138007.50	147151.00
Amphiregulin	597166.00	341586.50
Angiopoietin-1	82634.73	84149.00
Angiopoietin-like 4	81129.50	104607.62
ENPP-2/Autotaxin	84170.67	120777.50
Axl	54254.17	78759.50
BCL-x	92089.00	156523.50
CA125/MUC16	63029.50	82034.12
E-Cadherin	294947.50	385892.50
VE-Cadherin	194388.00	235845.56
CapG	636348.50	421598.00
Carbonic Anhydrase IX	114981.00	105756.00
Cathepsin B	245088.00	333878.00
Cathepsin D	1192311.00	1329912.50
Cathepsin S	165322.50	201248.00
CEACAM-5	170910.00	176950.00
Decorin	169087.00	224901.50
Dkk-1	112274.00	106604.50
DLL1	109348.73	151114.50
EGF R/ErbB1	148844.50	141227.00
Endoglin/CD105	102842.00	95199.56
Endostatin	229935.50	165155.00
Enolase 2	866167.50	618212.00
eNOS	18111.17	26614.00
EpCAM/TROP1	2014687.50	2425392.50
ERα/NR3A1	271080.00	288013.00
ErbB2	219567.50	330838.00
ErbB3/Her3	568899.50	689040.00
ErbB4	221432.00	306652.50
FGF basic	148672.50	136643.50
FoxC2	87906.50	89096.50
FoxO1/FKHR	561706.50	281970.00
Galectin-3	2138295.00	2397270.50
GM-CSF	56731.50	110538.56
CG α/β (HCG)	119785.00	142167.00
HGF R/c-Met	43770.50	45474.00
HIF-1α	111566.00	101622.00
HNF-3β	49385.84	53521.18
HO-1/HMOX1	194203.50	241828.50
ICAM-1/CD54	147682.00	118841.00
IL-2 Rα	53103.89	50798.50
IL-6	39251.34	34063.12

Analyte	DMSO(Control)	RGB-1
CXCL8/IL-8	82156.50	91471.68
IL-18 BPa	55145.45	66678.50
Kallikrein 3/PSA	84426.00	102179.00
Kallikrein 5	43029.90	53691.56
Kallikrien 6	48380.67	67355.00
Leptin	67451.45	81566.00
Lumican	41984.00	41327.56
CCL2/MCP-1	41590.73	47465.00
CCL8/MCP-2	64998.73	84997.68
CCL7/MCP-3	34040.84	52566.00
M-CSF	75350.50	67902.12
Mesothelin	107397.73	108852.00
CCL3/MIP-1α	58130.73	59189.50
CCL20/MIP-3α	68239.00	74989.00
MMP-2	82383.00	103147.00
MMP-3	100933.73	119920.00
MMP-9	44551.67	64024.00
MSP/MST1	66637.67	81112.00
MUC-1	648262.00	515830.50
Nectin-4	606805.50	389725.50
Osteopontin (OPN)	156343.00	178831.50
p27/Kip1	366679.50	1123511.00
p53	177072.00	108120.00
PDGF-AA	85661.73	76065.00
CD31/PECAM-1	47572.50	58118.50
Progesterone R/NR3C3	796330.00	681339.00
Progranulin	1277008.50	1577667.50
Prolactin	73192.84	105733.50
Prostasin/Prss8	650293.50	774893.50
E-Selectin/CD62E	41791.73	59125.50
Serpin B5/Maspin	410485.00	282996.50
Serpin E1/PAI-1	64850.45	83744.50
Snail	83859.50	91881.50
SPARC	90222.00	125404.00
Survivin	133703.73	56050.39
Tenascin C	42993.24	43526.06
Thrombospondin-1	114724.50	125133.50
Tie-2	82735.50	69533.00
u-Plasminogen Activator/Urokinase	63814.35	68060.00
VCAM-1/CD106	35373.17	33068.74
VEGF	67932.74	62054.50
Vimentin	38617.17	34374.36

Table S2. Protein expression levels of 84 cancer-related human genes. Data are shown by fold difference compared with DMSO controls.

Analyte	RGB-1/DMSO
α-Fetoprotein	1.066254
Amphiregulin	0.572013
Angiopoietin-1	1.018325
Angiopoietin-like 4	1.289391
ENPP-2/Autotaxin	1.434912
Axl	1.451676
BCL-x	1.699698
CA125/MUC16	1.301519
E-Cadherin	1.308343
VE-Cadherin	1.213272
CapG	0.662527
Carbonic Anhydrase IX	0.919769
Cathepsin B	1.362278
Cathepsin D	1.115407
Cathepsin S	1.217306
CEACAM-5	1.03534
Decorin	1.330093
Dkk-1	0.949503
DLL1	1.38195
EGF R/ErbB1	0.948822
Endoglin/CD105	0.925688
Endostatin	0.718267
Enolase 2	0.713733
eNOS	1.46948
EpCAM/TROP1	1.203855
ERα/NR3A1	1.062465
ErbB2	1.506771
ErbB3/Her3	1.211181
ErbB4	1.384861
FGF basic	0.919091
FoxC2	1.013537
FoxO1/FKHR	0.501988
Galectin-3	1.121113
GM-CSF	1.948451
CG α/β (HCG)	1.186851
HGF R/c-Met	1.038919
HIF-1α	0.910869
HNF-3β	1.083735
HO-1/HMOX1	1.245232
ICAM-1/CD54	0.804709
IL-2 Rα	0.956587
IL-6	0.867821

Analyte	RGB-1/DMSO
CXCL8/IL-8	1.113383
IL-18 BPa	1.209139
Kallikrein 3/PSA	1.210279
Kallikrein 5	1.247773
Kallikrien 6	1.392188
Leptin	1.209255
Lumican	0.984365
CCL2/MCP-1	1.14124
CCL8/MCP-2	1.307682
CCL7/MCP-3	1.544204
M-CSF	0.90115
Mesothelin	1.013541
CCL3/MIP-1α	1.018214
CCL20/MIP-3α	1.098917
MMP-2	1.252042
MMP-3	1.188106
MMP-9	1.437073
MSP/MST1	1.217209
MUC-1	0.795713
Nectin-4	0.642258
Osteopontin (OPN)	1.143841
p27/Kip1	3.064014
p53	0.610599
PDGF-AA	0.88797
CD31/PECAM-1	1.221683
Progesterone R/NR3C3	0.855599
Progranulin	1.23544
Prolactin	1.444588
Prostasin/Prss8	1.191606
E-Selectin/CD62E	1.414766
Serpin B5/Maspin	0.68942
Serpin E1/PAI-1	1.291348
Snail	1.09566
SPARC	1.389949
Survivin	0.419213
Tenascin C	1.012393
Thrombospondin-1	1.09073
Tie-2	0.840425
u-Plasminogen Activator/Urokinase	1.066531
VCAM-1/CD106	0.934854
VEGF	0.91347
Vimentin	0.890132

Table S3. Nucleotide sequences of 5'UTRs (wild-type and mutants) of Nectin-4 and CapG. Start codons are shown in bold. Potential G-quadruplex-forming sites are underlined.

Nectin-4 wild-type	5'-ACC UGU UCU GAC CUG CUG AGC AGG UUC CCA GGU UUC UGC CGU CGU UGU UGG<br>CCA CAG CGU GGG AAG CAG CUC UGG GGG AGC UCG GAG CUC CCG AUC ACG GCU UCU<br>UGG GGG UAG CUA CGG CUG GGU GUG UAG AAC GGG GCC GGG GCU GGG GCU GGG UCC<br>CCU AGU GGA GAC CCA AGU GCG AGA GGC AAG AAC UCU GCA GCU UCC UGC CUU CUG<br>GGU CAG UUC CUU AUU CAA GUC UGC AGC CGG CUC CCA GGG AGA UCU CGG UGG AAC<br>UUC AGA AAC GCU GGG CAG UCU GCC UUU CAA CCC AGG UAU ACA UAG UUG GAA AUU<br>CGC CAC C**AU G**-3'
Nectin-4 mutant1	5'-ACC UGU UCU GAC CUG CUG AGC AGG UUC CCA GGU UUC UGC CGU CGU UGU UGG<br>CCA CAG CGU GGG AAG CAG CUC UGG GGG AGC UCG GAG CUC CCG AUC ACG GCU UCU<br>UGG GGG UAG CUA CGG CUG GGU GUG UAG AAC AAA ACC AAA ACU AAA ACU AAA UCC<br>CCU AGU GGA GAC CCA AGU GCG AGA GGC AAG AAC UCU GCA GCU UCC UGC CUU CUG<br>GGU CAG UUC CUU AUU CAA GUC UGC AGC CGG CUC CCA GGG AGA UCU CGG UGG AAC<br>UUC AGA AAC GCU GGG CAG UCU GCC UUU CAA CCC AGG UAU ACA UAG UUG GAA AUU<br>CGC CAC C**AU G**-3'
CapG wild-type	5'-CUG AAU GGA GAA CAU GGC UUU CCU UGC UGG UCU CAG ACU CAG AGA UGC CCU<br>CCC UGA UUU GCC UUG GCU GUG ACC UGC AGC GGG CUG AGG CCA CUG UGG UGG GGC<br>UGU GUG GAC CUC CAG CCU CCC UGU GUG CCC CUC UGA GGA GAC GGC CUG GCA UAC<br>CCA CUG CCC ACC CCA GUG ACU GCU CUU CUG CUU CAG GCC UGC UGG CCU CCC AGC<br>ACU GCC UGC CCC UCC CUG UCG GGG GAC AUC GCC UCC ACA CCG GCU GGG GAA GGA<br>GCC CAG GGG UGG GGC UGG UGG GUG GGG CUG GUG GUU GGG GCA GCC AGA GAA GUA<br>AGA GGG AAG UGA GAA GCC GGG UGG GCA GGC UGG AAG GAA GAC GAA CCU ACG AAG<br>CAG AGA UCU GAA GAC AGC AAU UCG CCA CC**A UG**-3'
CapG mutant1	5'-CUG AAU GGA GAA CAU GGC UUU CCU UGC UGG UCU CAG ACU CAG AGA UGC CCU<br>CCC UGA UUU GCC UUG GCU GUG ACC UGC AGC GGG CUG AGG CCA CUG UGG UGG GGC<br>UGU GUG GAC CUC CAG CCU CCC UGU GUG CCC CUC UGA GGA GAC GGC CUG GCA UAC<br>CCA CUG CCC ACC CCA GUG ACU GCU CUU CUG CUU CAG GCC UGC UGG CCU CCC AGC<br>ACU GCC UGC CCC UCC CUG UCG GGG GAC AUC GCC UCC ACA CCG GCU GGG GAA GGA<br>GCC CAA AAA UGG GGC UGG UGG GUG GGG CUG GUG GUU GGG GCA GCC AGA GAA GUA<br>AGA GGG AAG UGA GAA GCC GGG UGG GCA GGC UGG AAG GAA GAC GAA CCU ACG<br>AAG CAG AGA UCU GAA GAC AGC AAU UCG CCA CC**A UG**-3'
CapG mutant2	5'-CUG AAU GGA GAA CAU GGC UUU CCU UGC UGG UCU CAG ACU CAG AGA UGC CCU<br>CCC UGA UUU GCC UUG GCU GUG ACC UGC AGC GGG CUG AGG CCA CUG UGG UGG GGC<br>UGU GUG GAC CUC CAG CCU CCC UGU GUG CCC CUC UGA GGA GAC GGC CUG GCA UAC<br>CCA CUG CCC ACC CCA GUG ACU GCU CUU CUG CUU CAG GCC UGC UGG CCU CCC AGC<br>ACU GCC UGC CCC UCC CUG UCG GGG GAC AUC GCC UCC ACA CCG GCU GGG GAA GGA<br>GCC CAG GGG UGG GGC UGG UGG GUG GGG CUG GUG GUU AAA ACA GCC AGA GAA GUA<br>AGA GGG AAG UGA GAA GCC GGG UGG GCA GGC UGG AAG GAA GAC GAA CCU ACG AAG<br>CAG AGA UCU GAA GAC AGC AAU UCG CCA CC**A UG**-3'
CapG mutant3	5'-CUG AAU GGA GAA CAU GGC UUU CCU UGC UGG UCU CAG ACU CAG AGA UGC CCU<br>CCC UGA UUU GCC UUG GCU GUG ACC UGC AGC GGG CUG AGG CCA CUG UGG UGG GGC<br>UGU GUG GAC CUC CAG CCU CCC UGU GUG CCC CUC UGA GGA GAC GGC CUG GCA UAC<br>CCA CUG CCC ACC CCA GUG ACU GCU CUU CUG CUU CAG GCC UGC UGG CCU CCC AGC<br>ACU GCC UGC CCC UCC CUG UCG GGG GAC AUC GCC UCC ACA CCG GCU GGG GAA GGA<br>GCC CAA AAA UAA AAC UGG UAA AUA AAA CUG GUG GUU AAA ACA GCC AGA GAA GUA<br>AGA GGG AAG UGA GAA GCC GGG UGG GCA GGC UGG AAG GAA GAC GAA CCU ACG AAG<br>CAG AGA UCU GAA GAC AGC AAU UCG CCA CC**A UG**-3'

Table S4. DNA and RNA oligonucleotides for CD experiments and K_D determination. The guanine-repeat sequences are highlighted in red, while the G-A mutations are shown in blue. The G-quadruplex forming sequences are underlined.

Entry	Sequence
Nectin-4	5'-GGGUGUGUAGAACGGGGCCGGGGCUGGGCUGGG-3'
Nectin-4 mutant1	5'-GGGUGUGUAGAACAAAACCAAAACUAAACUAAA-3'
CapG mutant1	5'-AAAAUGGGGCUGGUGGGUGGGGCUGGUGGUUGGGG-3'
CapG mutant2	5'-GGGGUGGGGCUGGUGGGUGGGGCUGGUGGUUAAAA-3'
CapG mutant3	5'-GGGGUAAAACUGGUAAAUAAAACUGGUGGUUAAAA-3'
DNA-DNA duplex	5'–TTTTTTTTTTTTTTTTT-3'/ 3'-AAAAAAAAAAAAAAAAA-5'
RNA-DNA duplex	5'-UUUUUUUUUUUUUUUUU-3'/ 3'-AAAAAAAAAAAAAAAAA-5'
